# Harnessing Photothermal Synergy of Cs_4_CuSb_2_Cl_12_/MoS_2_ Composite for Photothermoelectric Energy Harvesting and Small Power Application

**DOI:** 10.1021/acsami.5c04527

**Published:** 2025-06-24

**Authors:** Varun Sridhar, Chien-Ting Wu, Surojit Chattopadhyay

**Affiliations:** † Institute of Biophotonics, National Yang-Ming Chiao-Tung University, #155, Section 2, Li Nong Street, Beitou District, Taipei 112, Taiwan; ‡ National Applied Research Laboratories, Taiwan Semiconductor Research Institute, Hsinchu 300, Taiwan

**Keywords:** light harvesting, double perovskite, Cs_4_CuSb_2_Cl_12_, photothermal, photothermoelectric application

## Abstract

Photothermal (PT) and photothermoelectric (PTE) energy conversions are emerging research topics. Innovative material designs integrating energy harvesting and PT conversions are sought to drive these hybrid systems. Here, we report a study of a popular photovoltaic material Cs_4_CuSb_2_Cl_12_ (CCSC), which, when combined with bulk MoS_2_ into a rational composite, demonstrates exceptional PT/PTE energy conversion upon near-infrared (NIR) illumination for small power applications. Comprehensive structural, composition, and optical characterization of pure CCSC, MoS_2_, and their phase-pure mixtures helped us to optimize a composite that demonstrated a synergistic PT conversion. Thermal conductivity (*k*) modeling of the composite based on CCSC’s low (0.32 W m^–1^ K^–1^) and MoS_2_’s superior thermal conductivity (155 W m^–1^ K^–1^) with measured optical power absorption could explain the PT/PTE behavior. The 75% CCSC composite demonstrated a maximum temperature differential (Δ*T*) of ∼30 °C, and PTE voltage and current output of 500 mV and 105 mA, respectively, on a TE generator corresponding to a maximum power density of 3.26 mW cm^–2^ and efficiency of 7.26 × 10^–3^% under a 3000 K lamp at 450 mW cm^–2^. The optimized PTE device successfully charged a 40 mAh Li-ion battery within 180 s under 3000 K illumination. For NIR photodetection, the composites showed high responsivity under 808 and 980 nm lasers, with the 75% CCSC sample excelling at 808 nm with ∼9 × 10^2^ V W^–1^ and the 100% CCSC sample leading at 980 nm with ∼8.2 × 10^2^ V W^–1^.

## Graphical abstract

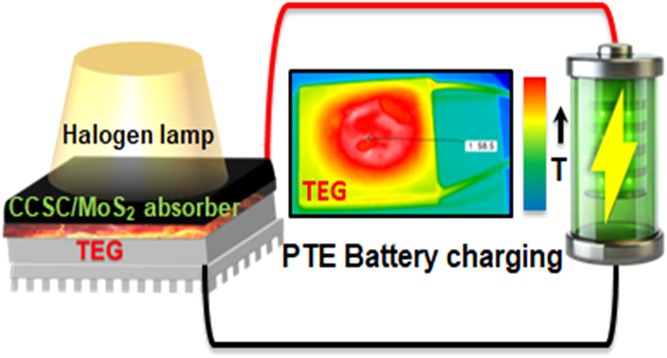

## Introduction

Energy conversion, such as photovoltaic (PV), photothermal (PT), and thermoelectric (TE), is essential to minimize losses and maximize utilization of abundant natural resources. While PV is already a commercial success, converting light into heat is an emerging renewable approach for energy harvesting, light detection, and PT-assisted energy management, such as storage. Artificial light harvesters (ALH) that absorb light and convert it into thermal energy are PT materials that enable applications such as photothermoelectric (PTE) energy generation/harvesting,^1–4^ PT-assisted near-infrared (NIR) photodetection,^5,6^ and energy storage.^7^ Recent advances in materials science have propelled the development of high-performance PT systems, but key challenges remain in achieving broadband absorption, optimizing thermal dissipation, and enhancing wavelength-specific functionalities. The thermoelectric (TE) effect is governed by the Seebeck effect which describes the generation of a voltage, Δ*V* = *S*Δ*T*, in a material due to a temperature gradient (Δ*T*), with *S* being the Seebeck coefficient, enabling direct conversion of thermal energy into electrical energy. When Δ*T* results due to illumination, a two-step energy conversion, we call it PTE energy conversion.

The PTE energy conversion, though promising, suffers from low efficiency due to its two-step process of PT heating, followed by the TE conversion. A wide variety of ALH materials have been explored for their use in PTE devices, ranging from transition metal dichalcogenides (TMDs), such as molybdenum disulfide (MoS_2_) and ReS_2_,^3,8^ to carbon-based materials such as carbon nanoparticles and carbon nanotubes.^2,9,10^ Hybrid materials, such as plasmonic nanoparticles combined with semiconductors, have also been reported for their ability to achieve broadband absorption, improve light-to-heat conversion, and for PTE applications.^5,10,11^ Meanwhile, TMDs exhibit unique optical properties, including tunable bandgaps and high light absorption across specific visible wavelength ranges,^12^ making them attractive candidates for PT conversion.^13^ Graphene’s exceptional thermal conductivity and charge transport properties have been employed to enhance the PT performance of PTE devices.^14,15^ However, graphene has a low and flat absorbance per layer over the broad spectral range.^16^ Among PT materials, two-dimensional (2D) transition metal dichalcogenides (TMDs) such as MoS_2_ have drawn significant attention for their tunable bandgap and thermal properties. Bulk MoS_2_, with a bandgap of ∼1.2 eV,^17^ exhibits strong absorption across the visible and NIR regions. Its high thermal conductivity (∼155 W m^–1^ K^–1^) ensures efficient heat dissipation,^18^ making it a promising candidate for PT applications. MoS_2_-based systems have demonstrated high PT conversion efficiencies in visible-light-driven energy generation and optical sensing. Although MoS_2_ absorbs NIR light, its performance in NIR-specific applications is limited due to suboptimal absorption characteristics in this region.

On the other hand, with their PV applicability and structural tunability, perovskites are already research hotspots and are now used for boosting the output of PTE devices.^4,19^ Through their integration into PTE systems, the perovskites have shown promise in addressing the challenges of thermal dissipation and wavelength-specific performance optimization. However, their performance over a broad range of wavelengths while retaining an acceptable efficiency is still lacking. Conventional perovskites, having a general formula ABX_3_ (A and B are cations, X is anion), are known to suffer from degradation, lacking long-term stability.^20,21^ Inorganic halide double perovskites, particularly microcrystalline Cs_4_CuSb_2_Cl_12_ (CCSC), have emerged as stabler candidates with broad absorption.^22^ They have also proved to be promising PT materials due to their high absorption coefficients.^23^ With a bandgap of ∼1.1 eV, CCSC strongly absorbs in the NIR spectrum, making it an ideal candidate for NIR absorption/detection and broadband light harvesting. However, the low thermal conductivity (∼0.32 W m^–1^ K^–1^) of microcrystalline CCSC films limits the heat flow challenging PTE applications.^23^ Additionally, the rough surface morphology and presence of voids hinder its PT and overall performance even further.^24^ Though the presence of voids, decreasing the packing fraction and limiting heat conduction, can be solved through the use of smaller CCSC crystals, their bandgap increases to ∼1.8 eV,^25^ limiting the performance in the visible-NIR region. So, despite their individual strengths, neither MoS_2_ nor CCSC alone fully addresses the need for a better ALH to suit PTE conversion. A facile physical mixture may offer a pathway to leverage superior thermal conductivity of bulk MoS_2_ and visible light absorption, complemented with CCSC’s strong NIR absorption and PT capabilities, to design an ALH for broad-spectrum PT/PTE applications.

PTE devices use TE generators (TEGs) to convert the heat generated by ALH into electricity. Both lab-made^3,26^ and commercial TEGs have been utilized,^1,2^ each having distinct advantages and limitations. Lab-made TEGs are highly customizable, allowing researchers to design configurations optimized for specific and multipurpose applications such as NIR detection and actuators.^10^ However, these systems often suffer from low conversion efficiencies limiting output power densities to <3 mW cm^–2^ despite using plasmonic nanostructures, unconventional perovskites, and MoS_2_, although they offer broader spectral coverage and flexibility.^3,10,19^ On the other hand, ALH-coated commercial TEGs have been shown to offer output power densities >8 mW cm^–2^,^1,2,4^ owing primarily to the increased number of p–n junctions and robust industrial manufacturing processes. Nevertheless, challenges persist in designing advanced ALH materials to achieve a large temperature gradient across the TEG for a higher output power suitable for consumer electronic applications. This approach incorporates secondary application possibilities for NIR photodetection.

The potential for NIR photodetection via PT-assisted PTE devices has garnered significant interest due to the demand for compact, efficient, and multifunctional systems. While multiple studies have explored the use of self-made PTE devices for NIR detection, these systems often rely on PT materials that serve dual roles as heat generators and TE elements, resulting in relatively lower output and limited responsivity, within 4.6 and 107 mA W^–1^,^5,10,19^ respectively. One of the few reported, using a commercial TEG, has shown NIR detection, achieving an output voltage of 14.5 mV under a 950 nm laser with a power density of 0.1 W cm^–2^, with an unknown efficiency/output power density/responsivity.^27^

This work explores the synergistic integration of CCSC and bulk MoS_2_ to create a multifunctional composite ALH for PTE-based energy generation, NIR (up to 980 nm) photodetection, and consumer electronic application in charging a Li-ion battery. We achieved enhanced PT performance by strategically combining CCSC’s efficient light absorption and low thermal conductivity with MoS_2_’s superior thermal conductivity and their combined broad spectral response. The PT performance translates to PTE power generation on commercial TEG enabling battery charging and strong NIR photodetection capabilities, particularly in the 808 and 980 nm wavelength regions.

## Materials and Methods

### Material Synthesis

Cs_4_CuSb_2_Cl_12_ (CCSC) microcrystals were synthesized following a slightly modified technique from the previously known method.^22^ 42.6 mg CuCl_2_·2H_2_O (98%, Emperor Chemical Co., Ltd., Taiwan), and 114 mg SbCl_3_ (99%, Alfa Aesar), were dissolved in 10 mL acetone in a centrifuge tube. 101 mg CsCl (>99%, Tokyo Chemical Industry Co., Ltd.) was dissolved in 100 μL DI water and added to the centrifuge containing CuCl_2_ and SbCl_3_ solution. The solution was then vortexed and sonicated consecutively for 60 s each until a brownish-black solution was formed without any yellow phase. This was further centrifuged at 5000 rpm, and the precipitate was collected and dried overnight at 45 °C in the oven.

### Device/Film Fabrication

As-purchased bulk MoS_2_ (98.5%, Acros Organics) and as-synthesized CCSC powder were taken in stoichiometric ratios (CCSC wt % = 0, 25, 50, 75, 87.5 and 100) making up to a total of 250 mg each, and ground in 500 μL isopropanol for 50 times using a mortar-pestle. This was drop coated on thermoelectric generator (TEG) modules (SP1848–27145 SA, 150 °C) and glass substrates for film characterization and photothermoelectric (PTE) studies.

### Material Characterization

Scanning electron microscope (SEM) images were captured using field emission SEM (FESEM, 6700F, JEOL, Japan), equipped with energy-dispersive X-ray spectroscopy (EDS) for elemental analysis. To understand the roughness profile, atomic force microscopy (AFM) was performed on MoS_2_/CCSC films coated on Si substrates by using a Veeco Dimension 3100 scanning probe microscope (D3100) in tapping mode. Transmission electron microscopy (TEM) was carried out on ground powders drop coated on Ni TEM grid (200 mesh, Ted Pella), employing JEOL JEM-2000EX. The high-resolution TEM (HRTEM) images were recorded using a JEOL (JEM 2010F, Japan) machine. X-ray diffraction (XRD) was performed on ground CCSC/MoS_2_ films by drop coating them on Si substrates, and measured under the illumination of 1.23 Å Synchrotron X-ray source (IU22, TPS beamline 09A) with a spot size of 400 × 700 μm^2^, using a Pilatus X-200X detector and a scintillation counter. The 2θ values in the XRD results were later converted to 1.54 Å. X-ray photoelectron spectroscopy (XPS, Thermo Fisher Scientific Theta Probe) was performed using a monochromated X-ray source (Al Kα, photon energy 1486.6 eV) focused on 400 × 500 μm^2^ elliptical spot on the MoS_2_/CCSC films coated on Si substrates. The XPS spectra were fitted with Gaussian–Lorentzian line shapes by using XPSPEAK41 software. The UV–vis absorption spectra were recorded using a spectrophotometer (JASCO V-770, Japan) in the range 400–1100 nm, with a resolution of 0.5 nm. The optical power transmission (*T*%) across the samples were measured upon coating identical films on glass substrates and illuminating those with 455 (B), 532 (G), 661 (R), 808 (equipped with a 20 mm collimator, MDL-N 18060582), and 980 nm (equipped with a 20 mm collimator, MDL-N 18057059 Changchun New Industries Optoelectronics Technology Co., Ltd., Taiwan), and measuring the power of the transmitted light. A combined illumination of the RGB lasers was considered as white light illumination. The incident laser powers were controlled using a graded neutral density filter and were measured directly with a calibrated detector (Ophir Nova II) equipped with a silicon photodiode (Ophir PD300-UV). The *T*% from the blank glass was subtracted as the background, and the *T*% from the samples was later converted into absorbance (1 – *T*%).

Differential scanning calorimetry (DSC) measurements were done with a low temperature (RT-200 °C) Netzsch 204F1 (Germany) machine using 3–10 mg powder samples. Pure sapphire was used as the standard (std.). The specific heat (*C*_p,sample_) of the samples were estimated using the eq 1

1 $$C_{p,\mathrm{sample}}=\frac{m_{\mathrm{std}.}\left(\mathrm{DS}C_{\mathrm{sample}}-\mathrm{DS}C_{\mathrm{baseline}}\right)}{m_{\mathrm{sample}}\left(\mathrm{DS}C_{\mathrm{std}.}-\mathrm{DS}C_{\mathrm{baseline}}\right)}C_{p,\mathrm{std}.}$$

where, *m* is mass, (DSC_sample_ – DSC_baseline_) and (DSC_std._ – DSC_baseline_) is the DSC output data for the samples and the standard, respectively. *C*_p,std._ = 0.785 J g^–1^ K^–1^ was used for sapphire. The PT studies were performed using a NIR thermal camera (IRM-P384A, Ching Hsing Computer Tech, Taiwan), by imaging the local temperature distribution of the surface of the as-fabricated PTE devices, with a temperature resolution of 0.1 °C and temporal resolution of 0.5 s. The temperatures mentioned in the PT images are the exact temperatures at the center of the illumination spot. The PTE measurements (current–voltage, current–time, and voltage–time) were performed using a Keithley 2540 source meter, equipped with a laboratory-assembled probe station, under light sources such as commercially purchased GU10 + C lamps with power density of 450 mW cm^–2^, 808 and 980 nm lasers using a 20 mm collimator, with various power densities.

## Results and Discussion

We studied the morphology of pure bulk MoS_2_ (indicated as MoS_2_ or 0% CCSC, unless otherwise specified), a ground admixture of CCSC/MoS_2_ (indicated as 25, 50, and 75% CCSC) and pure CCSC (100%) using scanning electron microscopy (SEM), and the images are shown in Figure 1a–e, respectively, where the pure MoS_2_ film (Figure 1f) displayed a smooth and continuous surface morphology,^28^ indicative of a compact film. When CCSC was sequentially added to the MoS_2_ in 25, 50, and 75 wt % (Figure 1b–1d), a progressive increase in the density of voids and pinholes was observed, which may arise as an artifact of fabrication conditions in addition to the mixing ratios. In contrast to the pure MoS_2_, the 100% CCSC films (Figure 1e) exhibited a rough surface morphology with a high density of voids and clustering. Such morphology of the CCSC films matched with the reported data.^24^ Further corroboration came from atomic force microscopy (AFM) of all of these films (Figure 1f–j). The root-mean-square (RMS) roughness in these films increased monotonically with increasing wt % of the CCSC (Figure 1k).

**Figure 1:**
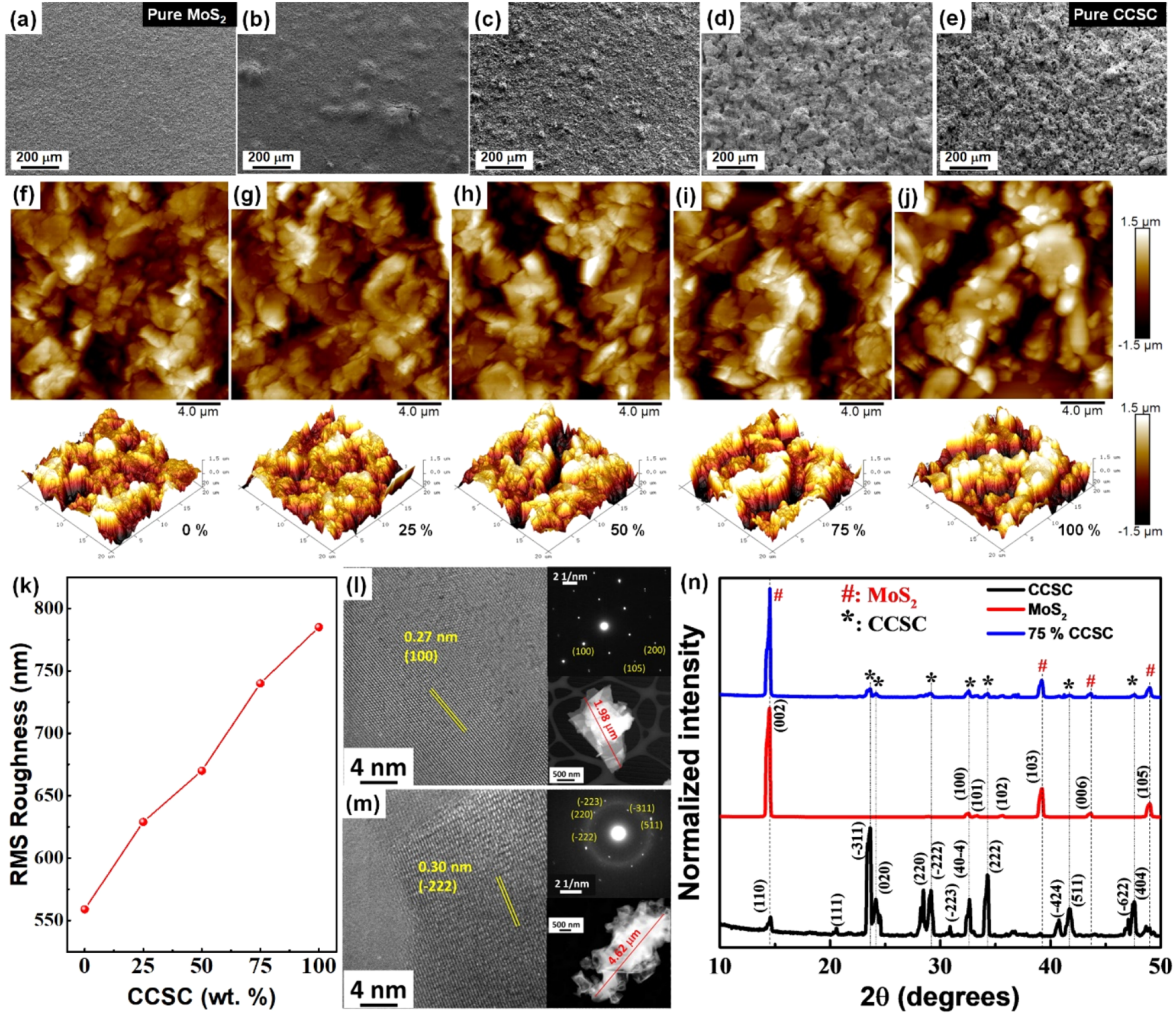
Morphology and structure of CCSC/MoS_2_ composite films. SEM images of (a) pure MoS_2_ film, and those with (b) 25, (c) 50, (d) 75% CCSC admixture, and (e) a pure (100%) CCSC film on Silicon substrate. The 2D (top) and the 3D (bottom) AFM roughness images of MoS_2_/CCSC films with (f) 0, (g) 25, (h) 50, (i) 75, and (j) 100% CCSC wt %, on Si substrates. (k) Variation of the RMS roughness with CCSC wt % obtained from the AFM images. The line joining the data points is a guide to the eye only. HRTEM images of pure (l) MoS_2_ and (m) CCSC samples with respective lattice spacing. The insets in panels (l, m) show the sample’s respective SAED patterns (top) and the HAADF TEM images (bottom). (n) XRD pattern of films with various CCSC/MoS_2_ ratios. The MoS_2_ and CCSC peaks are indexed, and the vertical dashed lines indicate the pure MoS_2_ and/or CCSC peaks contributing to the 75 wt % CCSC composite sample.

Energy-dispersive X-ray spectroscopy (EDS) was performed on the 0, 50, 75, and 100% films to understand the chemical composition upon grinding (Figure S1a–e, Supporting Information) and tabulated in Table S1 (Supporting Information). The EDS of the 0% sample (pure MoS_2_) shows a chemical formula of MoS_1.76_, similar to other reports.^29^ EDS confirms that the composite film showed an increase in the atom % of Cs, Cu, Sb, and Cl with the increase in theoretical wt % of CCSC, as expected (Table S1, Supporting Information). The composition of pure CCSC was calculated to be Cs_5.52_CuSb_2.08_Cl_11.42_, similar to the reported data.^22^ The composite films showed no change in the experimentally calculated chemical formulas of MoS_2_ and CCSC independently, indicating that the composite films contained a physical mixture of phase-pure compounds. The XPS scans of the same were recorded and are presented in Figure S2a–f. The scans were deconvoluted to show the contributions of each element and bonding configurations in the shaded areas in Figure S2. There were no significant shifts observed with various concentrations of the composite films, showing that CCSC and MoS_2_ were chemically intact within the film. The XPS-deduced composition (Table S2), which shows an increase in Cs, Cu, Sb, and Cl and a decrease in Mo and S, with increasing CCSC wt %, closely matched with the SEM-EDS-deduced composition (Table S1).

We studied the structural properties of MoS_2_ and CCSC using high-angle annular dark-field transmission electron microscopy (HAADF TEM), and high-resolution TEM (HRTEM), as shown in Figure 1l,1m, respectively. Figure 1l shows a *d*-spacing of 0.27 nm corresponding to the (100) plane of MoS_2_.^30^ The selected area electronic diffraction (SAED) pattern of the bulk MoS_2_ (top inset, Figure 1l) shows spots that are identified as originating from the (100), (105), and (200) planes.^31^ The HAADF TEM image of the multilayered MoS_2_ flakes, with lateral sizes ranging from ∼1–4 μm, was observed in the HAADF TEM (bottom inset, Figure 1m). The size and shape of bulk MoS_2_ are consistent with other reports.^32^ The HRTEM image of the pure CCSC is shown in Figure 1m, where the lattice spacing was identified to be 0.30 nm, corresponding to the (−222) plane, with the top inset showing the spotty SAED patterns originating from (−222), (220), (−223), (−311), and (511) lattice planes, indicating that the as-synthesized CCSC microcrystals are highly crystalline in nature.^33^ The HAADF TEM of the pure CCSC (bottom inset, Figure 1m) shows microcrystalline flakes with lateral sizes of ∼4.6 μm, comparable to the other published data.^33^ The higher density of voids and pinholes in the pure CCSC samples (Figure 1e,1j) can be attributed to the larger flake size of CCSC (Figure 1m), which hinders packing and creates voids between the flakes. This observation aligns with the hypothesis that larger flakes and voids contribute to increased surface roughness and structural discontinuities, as seen from the AFM studies in Figure 1k. Smaller MoS_2_ crystals would fill up these voids for compact film formation required for effective heat generation and transport.

The crystallinity of the 0, 75, and 100% CCSC films was further studied using XRD (Figure 1n). The pure MoS_2_ (0% CCSC) film shows XRD peaks at 14.50, 32.53, 33.33, 35.66, 39.24, 43.63, and 49.00°, corresponding to (002), (100), (101), (102), (103), (006), and (105) planes, respectively.^34^ The 100% CCSC films show XRD peaks at 14.59, 20.56, 23.63, 24.14, 28.47, 29.15, 30.88, 32.65, 34.26, 40.76, 41.72, 47.06, and 47.60°, corresponding to (110), (111), (−311), (020), (220), (−222), (−223), (40–4), (222), (−424), (511), (−622), and (404) indices, respectively, matching with reported data.^22,25^ The 75% CCSC showed peaks originating from MoS_2_ and CCSC without any observable shifts, indicating retention of individual crystallinity of MoS_2_ or CCSC upon grinding and/or coating of the composite film on a surface. The AFM, TEM, HRTEM, XRD, SEM, and EDS studies confirm that the composite films retained their phase-pure crystallinity; however, with increasing MoS_2_, the packing density increased, which can be advantageous for the PT properties of the composite. The optical properties of the CCSC/MoS_2_ composite films are shown in detail in Figure S3 (Supporting Information).

The PTE devices were fabricated as shown in Figure 2a–2c. Stepwise, the blank TEG module (Figure 2a) was cleaned by wiping the hot side using a lint-free tissue dipped in ethanol and then wiped with dry tissue to avoid stains. Then, various mixtures of CCSC/MoS_2_ composites (0–100 wt %) were ground in a mortar-pestle with isopropanol and then drop coated on the hot side of the module (Figure 2b). This was allowed to dry overnight, and then, the final PTE device (Figure 2a) was used for further measurements under a 3000 K household lamp. These PTE devices work based on PT, and TE (Seebeck effect) conversion.^4^

**Figure 2:**
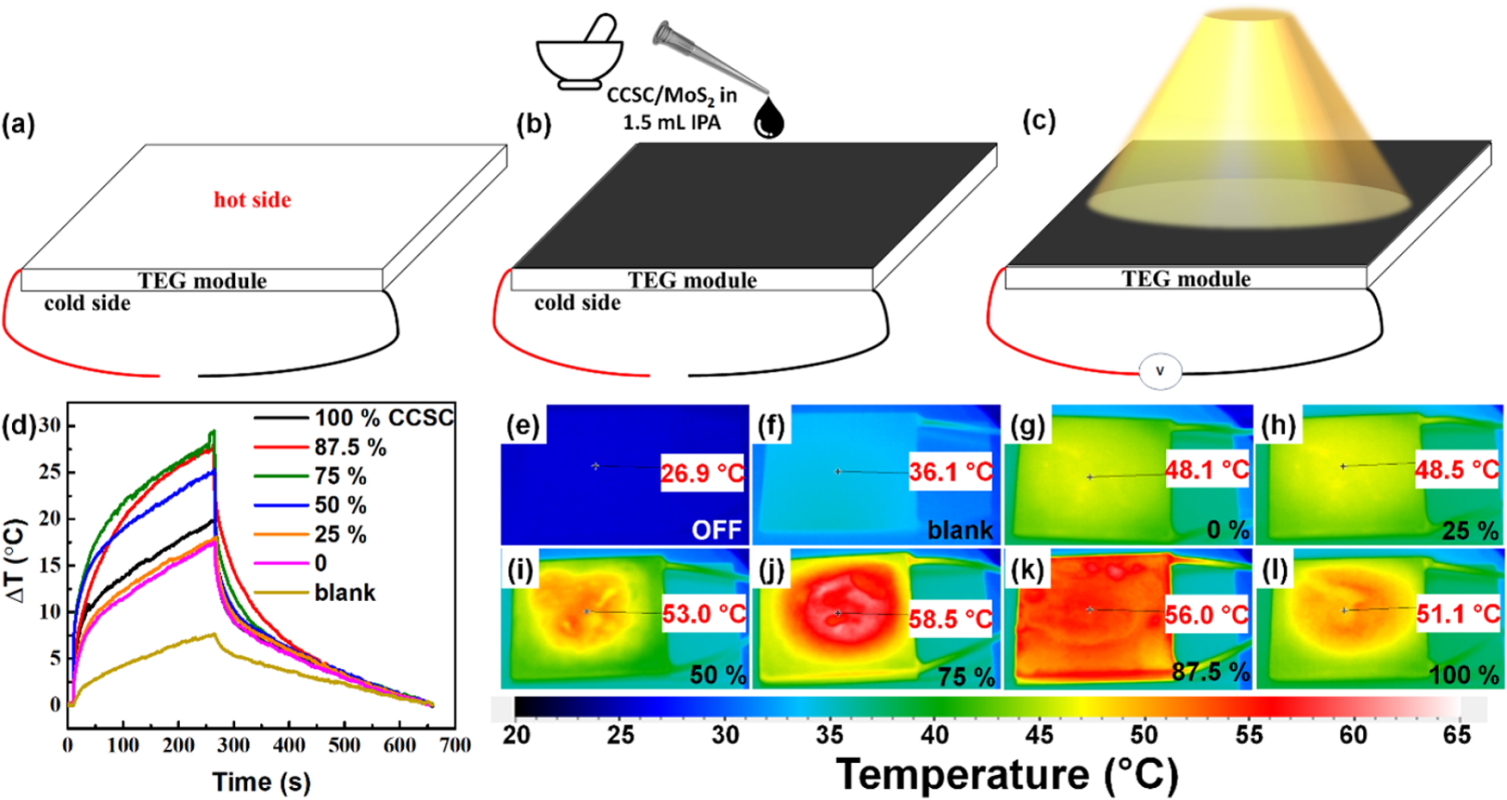
Schematic of the fabrication of CCSC/MoS_2_ composite photothermoelectric (PTE) devices and photothermal (PT) studies. (a) Blank thermoelectric generation (TEG) module, (b) drop coating a paste with various ratios of ground CCSC/MoS_2_ composite (in isopropanol/IPA), and (c) the final device under 3000 K lamp illumination (450 mW cm^–2^). (d) Dynamic PT response (Δ*T* vs time) of various CCSC/MoS_2_ composite-coated PTE devices under 3000 K illumination. (e) PT image of the PTE device under dark conditions. PT images of (f) blank, (g) 0, (h) 25, (i) 50, (j) 75, (k) 87.5, and (l) 100% CCSC composite-coated devices under the illumination of 3000 K lamp. The temperature at the arrowhead is mentioned in the white box.

The PT studies were performed on the various CCSC/MoS_2_ PTE devices under the illumination of a 3000 K lamp (450 mW cm^–2^), and a plot for the same is shown in Figure 2d. The blank device showed a Δ*T* < 10 °C. The pure MoS_2_ coated TEG sample (0% CCSC) showed a low Δ*T* of ∼15 °C compared to the 100% CCSC sample with a Δ*T* of ∼20 °C. Many composition variations were studied to confirm, beyond experimental errors, that the 75% CCSC sample did show the highest Δ*T* of ∼30 °C, followed by the 87.5 and 50% samples. PT images for the same are shown in Figure 2e–2l. The PT images for 0–100% CCSC samples (Figure 2g–2l) show Δ*T*, which follows the same trend as the PT plot shown in Figure 2d. The PT efficiency of the pure CCSC is better than the pure MoS_2_.^13,23^ However, the 75% CCSC composite-coated device showed a higher Δ*T*, which can be attributed to a combination of factors such as better compactness and thermal continuity in the film. Here, a contrasting thermal conductivity of MoS_2_ (155 W m^–1^ K^–1^)^18^ and CCSC (<0.32 W m^–1^ K^–1^)^23^ was used. Composites with contrasting thermal and electrical properties have shown better PT properties earlier.^5,35,36^ The PT synergy observed in the 75% CCSC composite arises from better optical absorption and thermal transport, aided by the improved compactness of the film.

We further characterized the CCSC/MoS_2_ composite-coated devices for their PTE properties in energy generation (Figure 3). The devices mentioned in Figure 2d were studied for their multicycle output voltage (*V*_out_) and output current (*I*_out_) under dynamic illumination of a 3000 K lamp (450 mW cm^–2^), as shown in Figure 3a. The blank device showed *V*_out_ and *I*_out_ values of 85 mV and 20 mA, respectively. The 0% CCSC (pure MoS_2_) device shows *V*_out_ and *I*_out_ of 294 and 69 mA, in contrast to the higher *V*_out_ and *I*_out_ of 440 and 96 mA of the 100% CCSC film. This is consistent with their UV–visible–NIR absorption. However, the 75% CCSC composite-coated device showed the highest *V*_out_ and *I*_out_ of 500 mV and 105 mA, respectively. To evaluate the PTE performance, we calculated the CCSC concentration-dependent device parameters such as estimated maximum output power (|*P*_max_|), maximum output power density (|*P*_max_| density), PTE efficiency (*h*), and responsivity (*R* in V W^–1^ and A W^–1^), similar to the reported parameters for PTE devices,^2,5,37^ using the eqs 2, 3, 4, 5, and 6, respectively

2 $$\left|P_{\max}\right|=V_{\mathrm{out}}\times I_{\mathrm{out}}\;\left(W\right)$$

3 $$\left|P_{\max}\right|\mathrm{density}=\left|P_{\max}\right|/A_{D}\;\left({W\;\mathrm{cm}}^{-2}\right)$$

4 $$\eta =\left|P_{\max}\right|/L_{I}$$

5 $$R=V_{\mathrm{out}}/\left[A_{D}\times L_{I}\right]\;\left({V\;W\;}^{-1}\right)$$

6 $$R=I_{\mathrm{out}}/\left[A_{D}\times L_{I}\right]\;\left({A\;W}^{-1}\right)$$

where *A*_D_, and *L*_I_, are device area (cm^2^), and light intensity or light power density (W cm^–2^), respectively. The |*P*_max_|, |*P*_max_| density, η, and *R* are shown in Figures S4a (Supporting Information), 4b, S4b (Supporting Information), and 3c, respectively. From the above calculations, we obtained the highest |*P*_max_|, |*P*_max_| density, η, *R* (from output voltage), and *R* (from output voltage) of 52.3 mW, 3.26 mW cm^–2^, 7.26 × 10^–3^%, 695 V W^–1^, and 145 A W^–1^, respectively for the 75% CCSC composite device. The output power (|*P*_max_|) density data confirmed from multiple studies show values >3σ (σ standard deviation) for the 75% sample than those nearest in value (Figure 3b), and attributed to the film’s enhanced PT conversion and transport due to a lower number of voids in the film.

**Figure 3:**
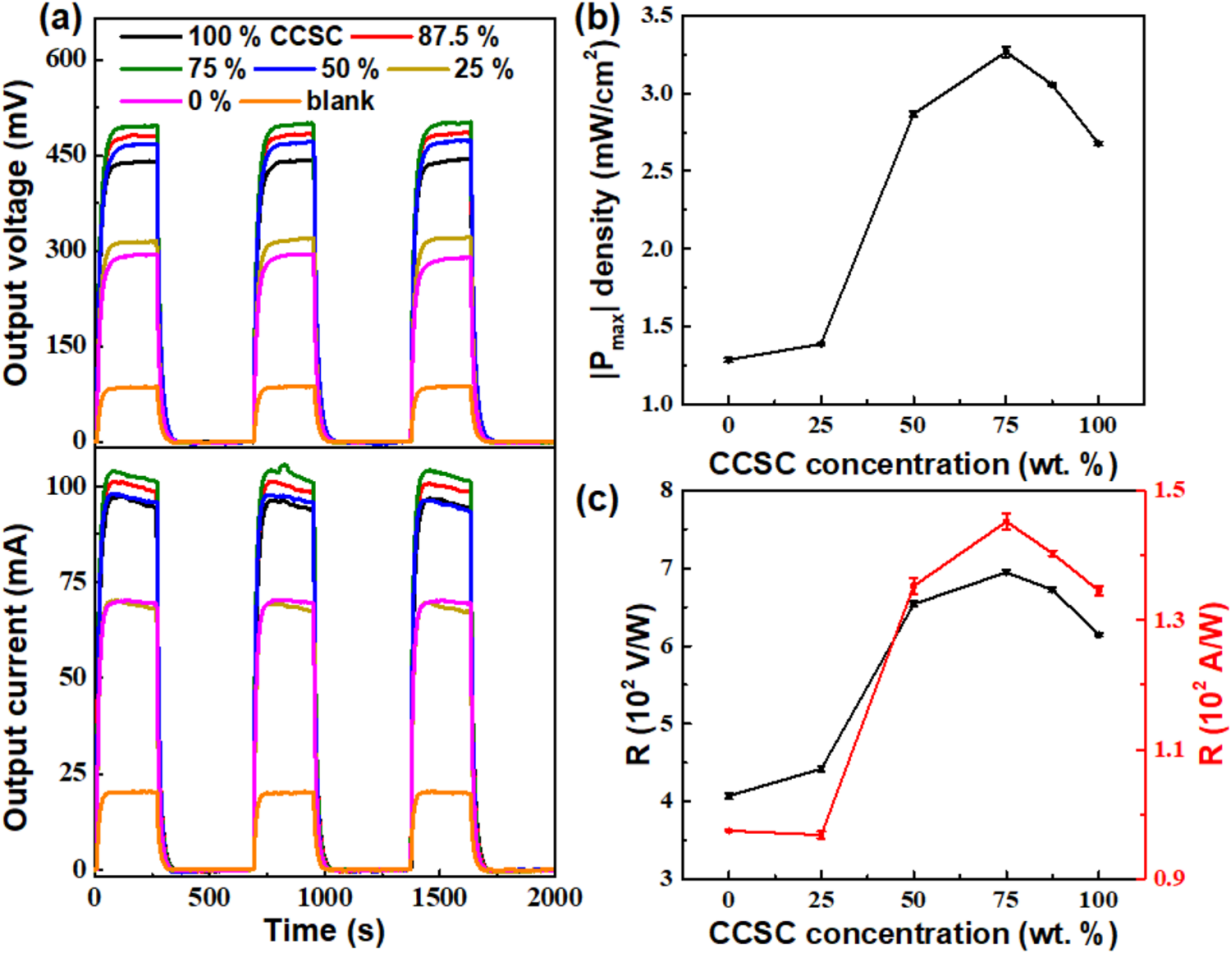
Photothermoelectric (PTE) characterization of the CCSC/MoS_2_ composite-coated devices under 3000 K light (450 mW cm^–2^). (a) Output voltage (top) and current (bottom) of various CCSC/MoS_2_ composite-coated PTE devices under illumination ON and OFF conditions, their (b) output power density, and (c) responsivities using current (left axis) and voltage (right axis) cycles. The error bars indicate standard deviations of 3 independent experiments. The line joining the data points is a guide to the eye only. The *P*_max_ density uses the area of the whole TEG device (4 × 4 cm^2^).

**Figure 4:**
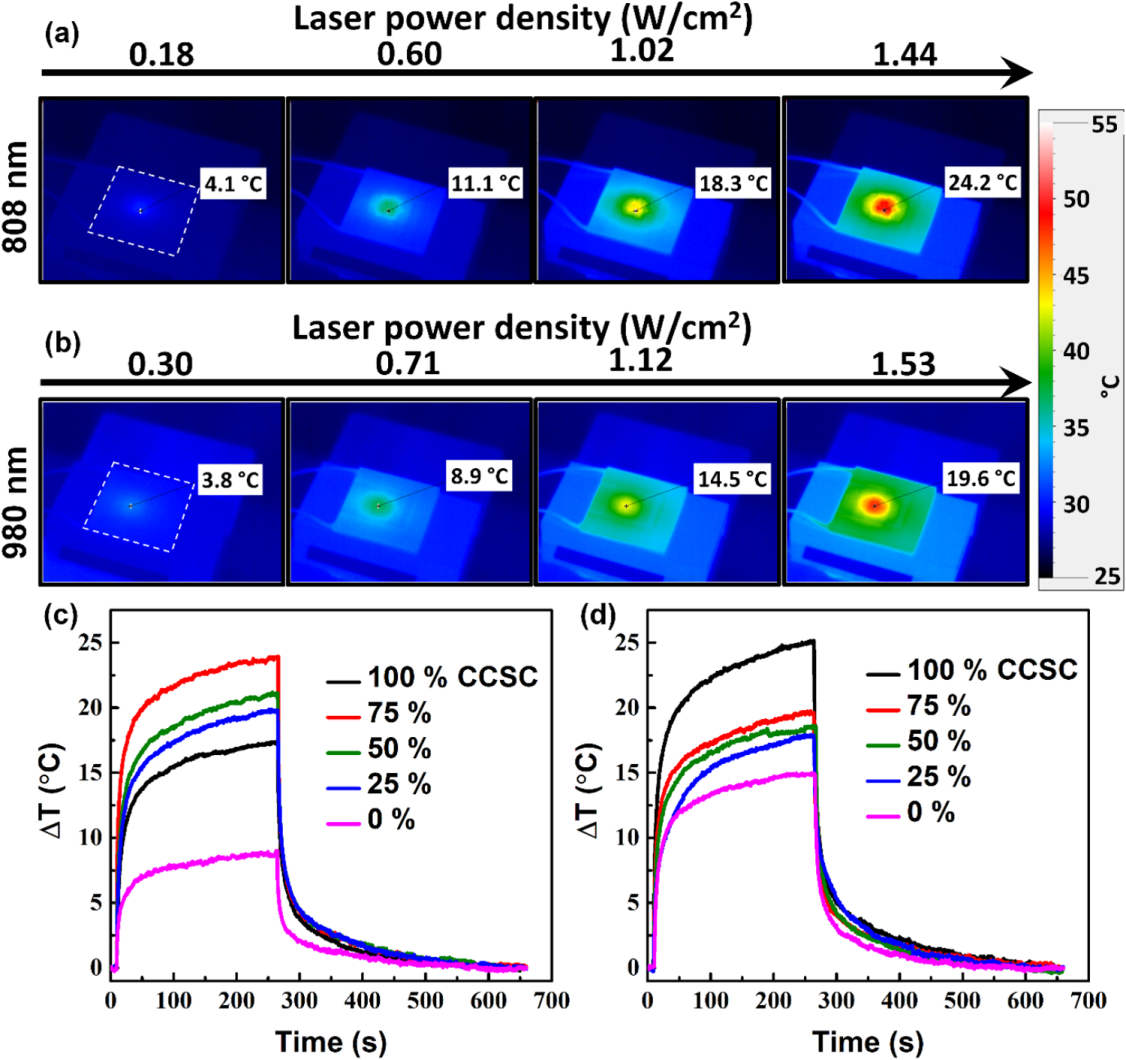
Photothermal (PT) studies of CCSC/MoS_2_ composite-coated photothermoelectric (PTE) devices. Power-dependent PT images of 75% CCSC-coated PTE device under (a) 808 and (b) 980 nm laser. The device area is outlined in (a) and (b). Temperature is color coded. Dynamic PT studies of various CCSC/MoS_2_ composite-coated PTE devices under (c) 808 and (d) 980 nm, under power densities of 1.44, and 1.53 W cm^–2^, respectively. The power density is estimated by using the area of the respective laser spots.

Furthermore, to obtain an idea about the NIR response of the CCSC/MoS_2_ composite-coated devices, we have characterized them under power-dependent 808 and 980 nm laser irradiation. The power-dependent PT images of the 75% CCSC-coated device are shown in Figure 4a,b. The power-dependent PT images for all of the CCSC/MoS_2_ composite-coated PTE devices under 808 and 980 nm lasers are shown in detail in Figures S5 and S6 (Supporting Information), respectively. The PT images show consistent power dependence, with a 75% CCSC-coated device showing the highest Δ*T* value under 808 nm illumination. A similar trend was observed under 3000 K illumination. However, under 980 nm illumination, the Δ*T* was highest for the 100% CCSC-coated device, followed in order by 75, 50, 25, and 0%. This trend can be attributed to 100% CCSC having a lower bandgap of 1.07 eV, compared to that of 1.22 eV of the bulk MoS_2_ (0% CCSC), where MoS_2_ barely absorbs 980 nm light. The PT trends (Δ*T* vs time) for the CCSC/MoS_2_ composite-coated devices under 808 and 980 nm illumination (under power densities of 1.44, and 1.53 W cm^–2^, respectively) are shown in Figure 4c,4d.

To understand the mechanism behind the PTE response in these films, we model the morphology of the composite films based on the observation from SEM and AFM studies (inset, Figure 5a) using CCSC, MoS_2_, and air as constituents. Here, the 0% film (pure MoS_2_) exhibits a good packing fraction and smoother surface in contrast to the 100% film (pure CCSC) that has significant void fraction (air gaps). We have calculated the absorbed power (*P*_abs_%) across the films under various laser illumination (Figure S7), using the measured transmitted power (*T*%) from eqs S(1) and S(2) in Supporting Information. The Δ*T* (under warm lamp illumination) varied in a way similar to that of the *P*_abs_ under combined RGB illumination and reached a maximum for the 75% CCSC film (Figure 5a). This variation is similar for individual R, G, B, and 808 nm illumination (Figure S7). However, for the 100% film, the presence of the optically inactive voids decreased the net absorption under all illuminations up to 808 nm and results in a drop in the Δ*T* and the resultant PTE outputs. Only for the 980 nm illumination did Δ*T* scale with *P*_abs_, throughout the range of the samples (Figure 5b). We attribute it to the high 980 nm absorption in CCSC compared to that in MoS_2_.^22,38^ We have estimated the effective thermal conductivity (*k*) in these composites using two standard models (Series and the Maxwell–Eucken configuration),^39^ assuming the published *k* values for pure CCSC and MoS_2_ as 0.32,^23^ and 155 W m^–1^ K^–1^,^18^ respectively (Figure 5c). The nature of the *k*-variation for the Series and ME model (Figure 5c) bears clear inverse correlation with the Δ*T* (Figure 5a,b). A lower *k* would imply a lower specific heat C_p_ (*k* = αρ*C*_p_, where α is the thermal diffusivity and ρ is the density), other factors remaining the same. A lower *C*_p_ would imply a quicker rate of PT heating and a larger Δ*T* under irradiation similar to that observed here. Using DSC, we have measured the *C*_p_ values for the samples (Figure S8) that showed pure MoS_2_ and CCSC at the higher and lower bounds, respectively, with the composites having values within these limits. Using published data for α and ρ in the pure films (CCSC, and MoS_2_),^18,23^ we could estimate the measured *k* in these films (Figure 5c) which matched closely with the model values to impart credibility to these models and the measurement.

**Figure 5:**
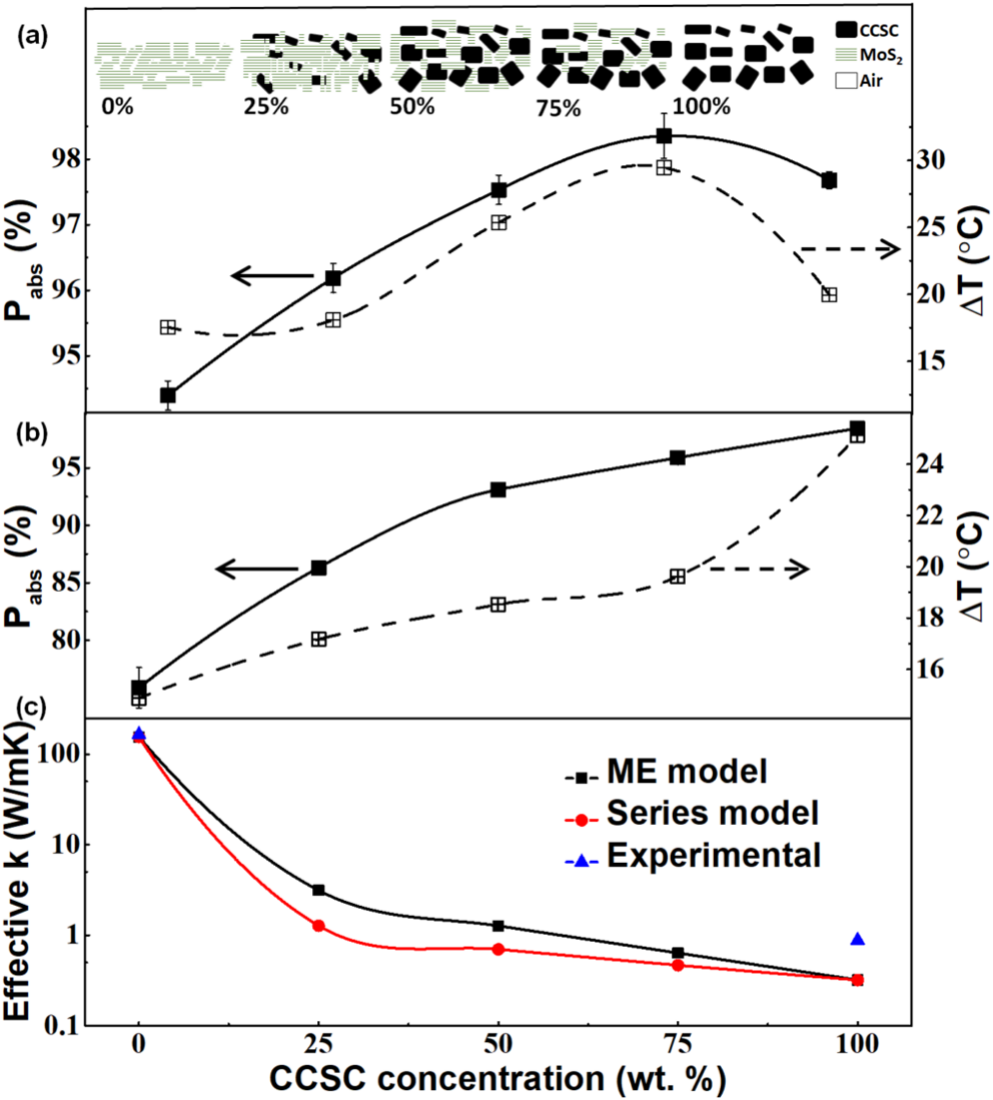
Photothermal properties of MoS_2_/CCSC films. (a) Variation of absorbed powers (*P*_abs_), under combined RGB (white) laser illumination for different CCSC wt %. Inset shows the schematic of the composite films and their constituents. (b) Variation of absorbed powers (*P*_abs_), under 980 nm collimated laser illuminations with CCSC wt %. Variation of the photothermal Δ*T* (right axis) under representative (a) warm lamp, and (b) 980 nm illumination is shown for reference. (c) Estimated effective thermal conductivity (*k*) of the various MoS_2_/CCSC composite films following the Maxwell–Eucken (ME), and Series configuration models. Measured *k* values for the 0 and 100% samples are shown. Error bars indicate scatter over three independent measurements. The lines joining the data points are guides to the eye only.

The PTE properties of the CCSC/MoS_2_ composite-coated devices were studied under 808 and 980 nm laser illuminations (Figure 6). The power-dependent PTE *V*_out_ and *I*_out_ cycles under the dynamic illumination of 808 nm (1.44 W cm^–2^) are shown in Figure 6a, where the highest values of *V*_out_ and *I*_out_ were obtained for the 75% CCSC device, following their PT response. The laser power-dependent |*P*_max_| density and *R* (V W^–1^) are shown in Figure 6b,6c. The |*P*_max_| density increases with the input laser power density (Figure 6b), similar to other PTE works.^19^ However, the *R* value remains almost flat over the laser power band used. The highest *R* of 9 × 10^2^ V W^–1^ was obtained for the 75% CCSC device under 808 nm illumination (1.44 W cm^–2^). The *V*_out_ and *I*_out_ under dynamic illumination of 980 nm (1.53 W cm^–2^) are shown in Figure 6d. The 100% CCSC shows the highest *V*_out_ and *I*_out_, following the trend of PT studies. The |*P*_max_| density and R (V W^–1^) are shown in Figure 6e,6f. The |*P*_max_| density increases with the input laser power density (Figure 6e) and the *R* (V W^–1^) remains flat over the measured powers (Figure 6f). The highest *R* of 8.2 × 10^2^ V W^–1^ was obtained for the 100% CCSC device under 980 nm illumination (1.53 W cm^–2^).

**Figure 6:**
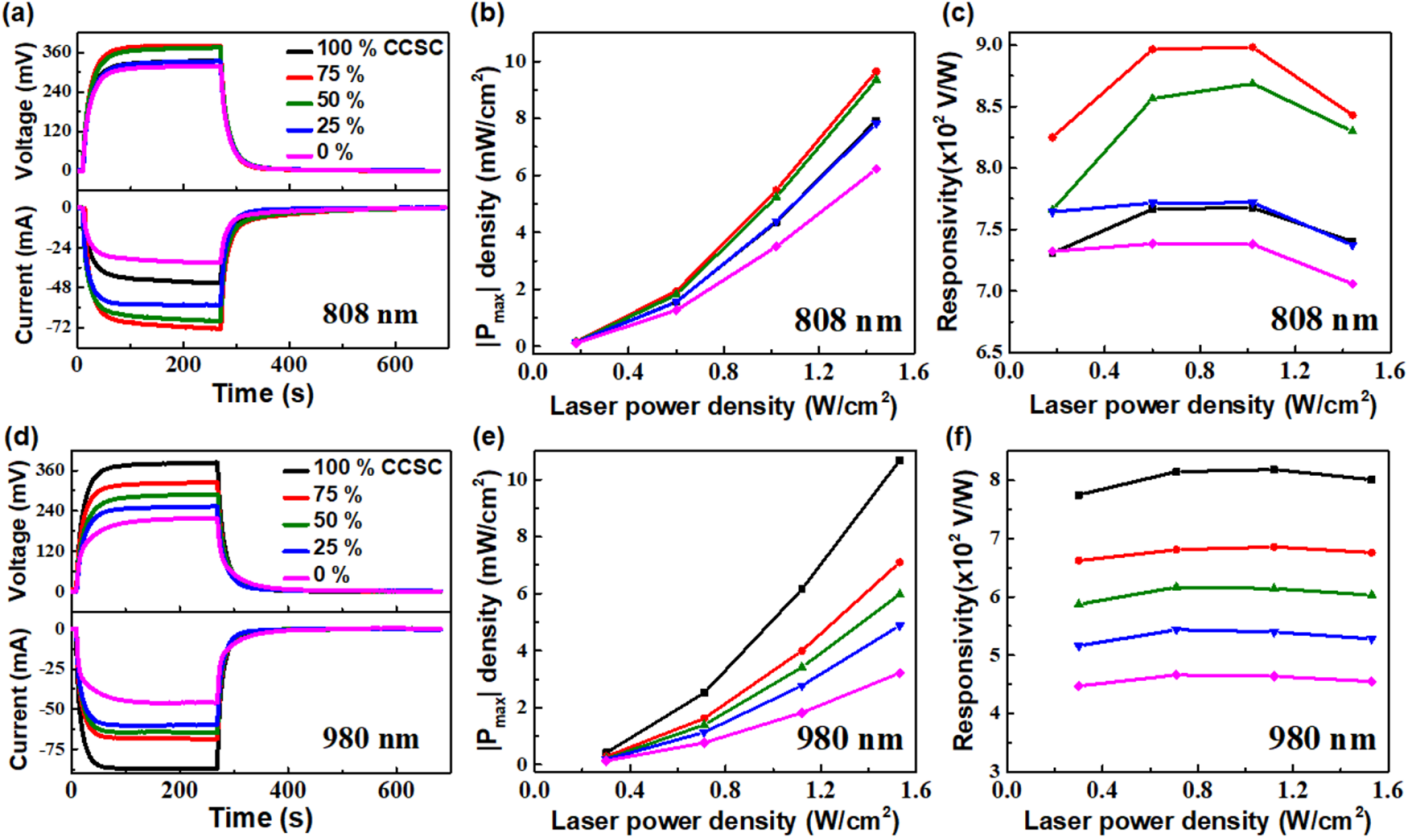
Photothermoelectric (PTE) characterization of CCSC/MoS_2_ composite-coated devices. Voltage–time (*V*–*t*) and current–time (*I*–*t*) characteristics of CCSC concentration-dependent coated PTE devices under (a) 808 nm illumination. Estimated (b) PTE |*P*_max_| density and (c) responsiveness as a function of 808 nm laser power density. Corresponding (d) *V*–*t*, Δ*I*–*t*, (e) PTE |*P*_max_| density, and (f) responsivity, as a function of 980 nm laser power density. The PTE |*P*_max_| density and laser power densities are calculated using the device and laser spot area, respectively. The lines joining the data points are guides to the eye only.

Although we have obtained the output characteristics of the CCSC/MoS_2_ PTE devices, we further characterized them for their Δ*I*–*V* and Δ*I*–*t* under bias conditions to check their functionality as PTE-based photodetectors. The laser power density-dependent Δ*I*–*V* characteristics of 0, 25, 50, 75, and 100% CCSC devices are shown in Figure S9a–e (Supporting Information). The photocurrent (Δ*I*) increased with increasing laser power density in all cases. However, the Δ*I* was highly asymmetrical in the case of the 0% CCSC (pure MoS_2_) device. The asymmetry increases with the increasing laser power density. This asymmetry is due to the combination of both Seebeck’s and Peltier’s effect, which are both inherent property of TEGs,^40^ where the device shows a temperature gradient at both sides of the device upon application of a bias voltage, as well as a power generated due to the applied temperature gradient between the hot and the cold surfaces. However, the asymmetry decreased in the case of CCSC/MoS_2_ composite devices with increasing CCSC concentration, which could be due to the lower thermal conductivity of the CCSC compared to that of the MoS_2_, and the higher photocurrent values offered by the devices with increasing CCSC concentration. From the Δ*I*–*V* curve, we obtained the Δ*I* values under the illumination of 808 nm (1.44 W cm^–2^) with 0, and 1 V bias, and shown them against the CCSC concentration in Figure S9f (Supporting Information), where we observe a higher Δ*I* in case of the 1 V bias, following the Δ*I*–*V* curve (Figure S9a–e, Supporting Information).

We then measured the bias-dependent photocurrent (Δ*I*) for the devices under dynamic illumination of an 808 nm laser and calculated their performance parameters, which are shown in Figure S10 (Supporting Information). The laser power density-dependent Δ*I*, and *R* (A W^–1^) under 0 V bias are shown in Figure S10a,b (Supporting Information), respectively. The Δ*I* is linear against laser power density, while the *R* remains flat over the measured laser power densities. Similar study is shown with a 1 V bias in Figure S10c,d (Supporting Information), where the Δ*I*, and *R* (A W^–1^) have increased in comparison to the 0 V bias, which is consistent with the Δ*I*–*V* curve in Figure S9 (Supporting Information).

We measured the Δ*I*–*V* under various power densities of 980 nm laser illumination, as shown in Figure S11a–e (Supporting Information) for devices with 0–100% CCSC concentrations, where the 100% CCSC shows the highest Δ*I*. The CCSC concentration-dependent Δ*I* values under 980 nm illumination (1.53 W cm^–2^) with 0, and 1 V bias are shown in Figure S11f (Supporting Information), where the Δ*I* increases as the CCSC concentration increases, and the Δ*I* values for all of the cases are higher in the case of 1 V bias in comparison to the 0 V bias condition. The laser power density-dependent performance parameters for the CCSC/MoS_2_ composite-coated devices under 980 nm laser illumination are shown in Figure S12 (Supporting Information), with calculations that are similar to those in Figure S10 (Supporting Information). The Δ*I* increases linearly with laser power density and is higher in the case of 1 V bias (Figure S12a,c, Supporting Information). The laser power density-dependent *R* remains flat and is higher in the case of 1 V bias in comparison to the 0 V bias condition (Figure S12b,d, Supporting Information). The study under laser illumination and bias conditions shows that these devices can be used in multipurpose applications such as energy generation and photodetection, with tunability of their responsivity according to the illumination type.

For a comparison to understand where our device output stands, we show the output voltages of various PTE devices reported across various input power densities in Figure 7, where the devices fabricated by using commercial TEGs are shown as blue spherical symbols and the ones using self-made TEGs are shown as pink rhombus symbols. The reported perovskite-based devices are marked with red circle as a reference for comparison with our device, where our device has so far shown the highest output among the perovskite-based PTE devices. The details of the same are shown in Table S3 (Supporting Information), which includes the year of publication and the light source used.

**Figure 7:**
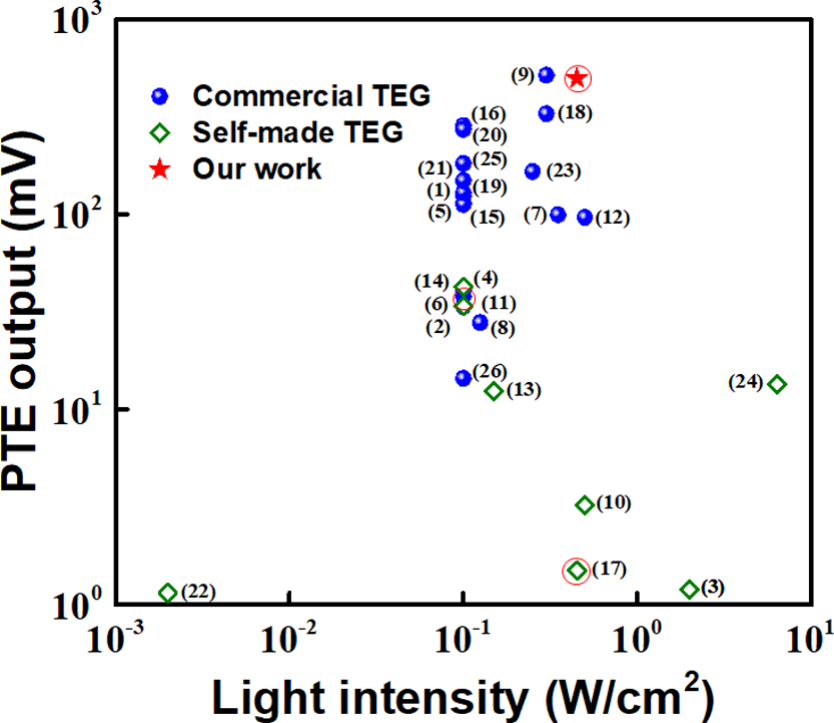
Comparison of the maximum output voltages reported from different ALH-coated PTE devices (commercial and self-made) and this work. The data from perovskite-based ALHs are circled in red. The details of illumination and device type are illustrated in Table S3 (Supporting Information). The numbers in parentheses indicate the source references of the data points tabulated in Table S3 and listed in Supporting Information.

Upon obtaining the PTE performance parameters from various types of illumination, we chose 3000 K, and used the optimum 75% CCSC PTE device to charge a 40 mAh Li-ion battery (3.7 V), which is shown in Figure 8. The battery charging setup is shown in Figure 8a, which consists of the optimum CCSC/MoS_2_ composite-coated PTE device illuminated by the 3000 K lamp (450 mW cm^–2^) that is connected as an input to a simple electronic charging board which steps up the PTE device’s voltage to charge the battery connected at the BAT (battery) terminals. An LED is used as an indicator that turns on only when the battery is fully charged, while the PTE device is still supplying the voltage to the charging board.

**Figure 8:**
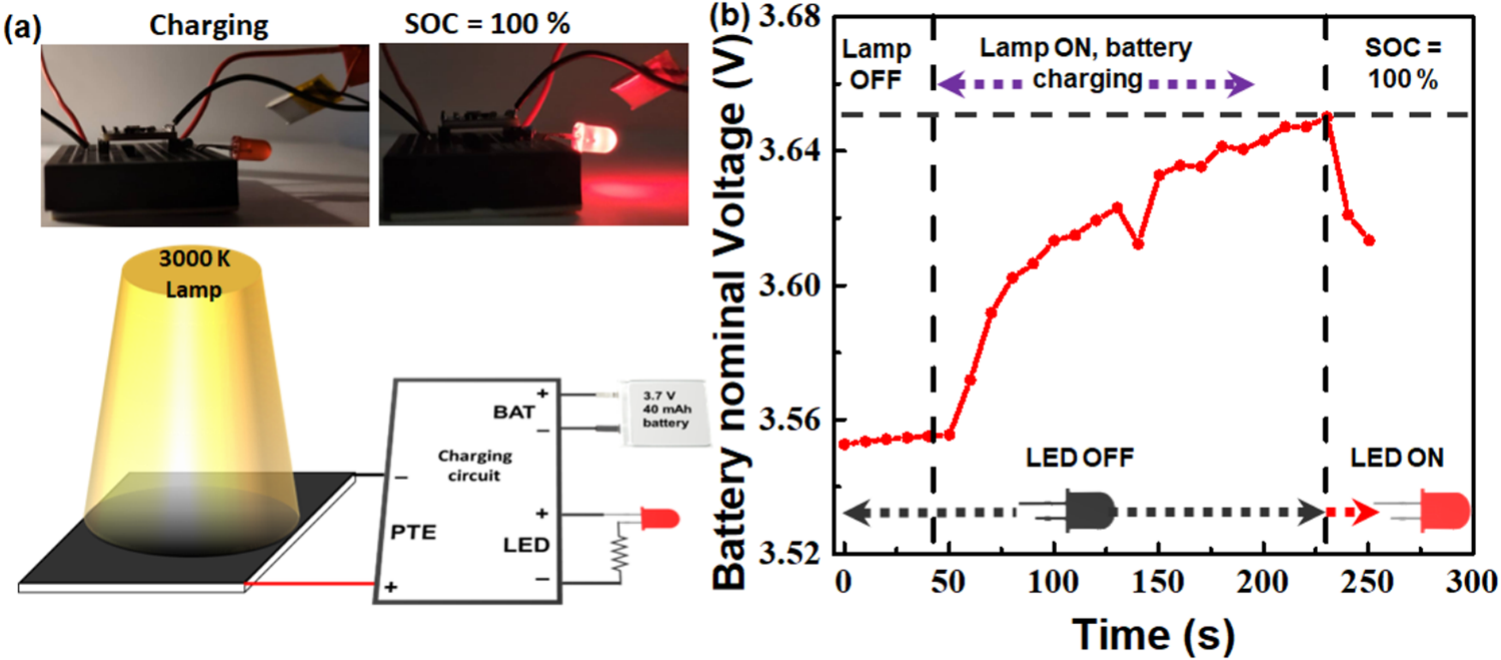
Battery charging application using PTE devices. (a) Photographic images (top) and schematic illustration (bottom) of a 3.7 V Li-ion battery (BAT) charging setup via light harvesting using the optimally coated PTE device. A 100% state of charge (SOC) turns ON the light emitting diode (LED), otherwise it is OFF. (b) Dynamic nominal voltage across the battery measured during charging using the 75% CCSC/MoS_2_ composite-coated PTE device under 3000 K light illumination (450 mW cm^–2^). The line joining the data points is a guide to the eye only. The insets show photographic images of the charging setup with SOC < 100% (during charging with lamp ON; indicator LED off), and at SOC = 100% (indicator LED on). The horizontal dashed line at 3.65 V indicates a 100% charged state for the battery. The vertical dashed line indicates the time the LED turns on (100% battery charge).

The battery was predischarged to 3.55 V and connected to its respective terminals charging board, and its voltage was monitored every 10 s thereafter (Figure 8b). After 50 s, the 3000 K lamp (450 mW cm^–2^) was turned on and the battery voltage started increasing, while the LED is still off. At 230 s (elapsed time after the charging started: 180 s), the battery reaches ∼3.65 V, and the LED turns on, which indicates that the battery has been charged to 100% of its state of charge, as shown in the supporting video file (sped 10 times). This study shows that the harvested light from the PTE can be further used for power generation and storage applications under household lighting.

## Conclusions

This study demonstrates the facile synthesis of a CCSC perovskite and bulk MoS_2_ composite as artificial light harvesters (ALH) for integrated photothermal (PT) energy conversion and photothermoelectric (PTE) voltage generation for low-power consumer electronic applications. Comprehensive structural, optical, and compositional characterization confirmed the composite components’ phase purity with better packing fraction than the pure films. The specific heats of the composite films demonstrated values within the upper (∼3.5 J/gK) and lower (0.8 J/gK) limits traced for pure MoS_2_ and CCSC, respectively, within 50–70 °C. The combination of CCSC’s efficient NIR absorption and low thermal conductivity with MoS_2_’s high thermal conductivity enabled optimized PT properties for this ALH. The 75% CCSC (25% MoS_2_) composite ALH achieved a significant Δ*T* (∼30 °C) under 3000 K illumination, resulting in PTE performance marked by a high output voltage, PTE output power density, and efficiency of 500 mV, 3.26 mW cm^–2^, and 7.26 × 10^–3^%, respectively. A clear mechanism was established from the direct measurement of absorbed power that corroborated the PT temperature gradient and resultant photothermoelectric outputs. A practical application was demonstrated by successfully charging a 40 mAh lithium-ion battery from 3.55 to 3.65 V within 180 s using the ALH-coated TE generator under 3000 K illumination. For NIR photodetection, the optimized ALH, with the 75% CCSC sample, excelled at 808 nm with a responsivity of 9 × 10^2^ V W^–1^, and the 100% CCSC sample leading under 980 nm with a responsivity of 8.2 × 10^2^ V W^–1^, correlating with CCSC’s narrower bandgap compared to that of the bulk MoS_2_. These findings emphasize the ability of CCSC/MoS_2_ composites to address key challenges in PT device performance, including broadband optical absorption, thermal management for efficient utilization of waste heat and light, and wavelength-specific functionalities.

## Supplementary Material





## Notes

Tables for CCSC/MoS_2_ composition and comparison of PTE devices, figures for optical, photothermal, and photothermoelectric characterization parameters for CCSC/MoS_2_ devices in detail (PDF)
Battery charging and its LED indication for its 100% SOC (MP4)
