# Supplementary material for: Harnessing Photothermal Synergy of Cs4CuSb2Cl12/MoS2 Composite for Photothermoelectric Energy Harvesting and Small Power Application
Source: ACS Appl Mater Interfaces. 2025 Jun 24;17(27):39584–94. doi: 10.1021/acsami.5c04527 (PMC12257448; doi:10.1021/acsami.5c04527)
Supplement: Supplementary file 1 [file am5c04527_si_001.pdf]

Supporting Information

Harnessing photothermal synergy of $Cs_4CuSb_2Cl_{12}/MoS_2$ composite for photothermoelectric energy harvesting and small power application

Varun Sridhar[ξ], Chien-Ting Wu[†], and Surojit Chattopadhyay[ξ]*

[ξ]Institute of Biophotonics, National Yang-Ming Chiao-Tung University, #155, Section 2, Li Nong Street, Beitou District, Taipei 112, Taiwan.

[†]Nano Device Materials Characterization Division, National Nano Device Laboratories, Hsinchu 300, Taiwan.

*Corresponding author: sur@nycu.edu.tw

Table S1. SEM-EDS-deduced chemical composition of the various CCSC/MoS_2 composite coated films.

CCSC (wt. %)	Mo	S	Cs (at. %)	Cu	Sb	Cl	Experimental chemical formula (Ref: $MoS_2/Cs_4CuSb_2Cl_{12}$)
0	36.2	63.8	-	-	-	-	$MoS_{1.76}$
25	33.8	61.7	1.6	0.1	0.4	2.4	$MoS_{2.33}/Cs_{16}CuSb_4Cl_{24}$
50	28.0	50.2	5.8	1.5	1.8	12.7	$MoS_{1.79}/Cs_{3.86}CuSb_{1.20}Cl_{8.46}$
75	16.6	26.6	14.5	3.0	6.1	33.1	$MoS_{1.60}/Cs_{4.83}CuSb_{2.03}Cl_{11.03}$
100	-	-	27.6	5.0	10.4	57.1	$Cs_{5.52}CuSb_{2.08}Cl_{11.42}$

Table S2. XPS-deduced chemical composition of the various CCSC/MoS_2 composite coated films.

**CCSC (wt. %)**	**Mo**	**S**	**Cs**	**Cu**	**Sb**	**Cl**	**Experimental chemical formula (Ref: $MoS_2/Cs_4CuSb_2Cl_{12}$)**
	**(at. %)**						
0	31.8	68.1	0	0	0	0	$MoS_{2.13}$
25	25.2	46.7	5.3	2.0	2.5	18.0	$MoS_{1.85}/Cs_{2.61}CuSb_{1.23}Cl_{8.81}$
50	21.5	40.5	5.9	2.3	4.3	25.2	$MoS_{1.88}/Cs_{2.57}CuSb_{1.89}Cl_{10.94}$
75	14.1	30.4	12.0	3.1	4.9	35.1	$MoS_{2.14}/Cs_{3.82}CuSb_{1.58}Cl_{11.17}$
100	0	0	21.5	5.0	12.0	61.3	$Cs_{5.52}CuSb_{2.08}Cl_{11.42}$

Table S3. Comparison of our work with various PTE device output voltages reported under various power densities and light sources.

Light intensity (W cm^{-2})	PTE Voltage (mV)		Light source	ΔT (°C)	Year	Ref.
	Commercial TEG	Lab-made TEG				
0.1	124	-	Solar	17.7	2017	1
0.1	33.9	-	Solar	20.9	2017	2
2	-	1.2	808 nm laser	45.0	2018	3
0.1	-	42.7	Solar	428.1	2018	4
0.1	115	-	Solar	5.0	2018	5
0.1	38	-	Solar	50.8*	2019	6
0.35	100	-	Solar	43.0*	2019	7
0.125	28	-	Solar	37.1*	2020	8
0.3	520	-	Solar	44.9	2020	9
0.5	-	3.24	IR lamp	160.1*	2021	10
0.1	-	34	Solar	36.7	2021	11
0.5	97	-	Solar	29.8*	2021	12
0.15	-	12.5	808 nm laser	28.0	2022	13
0.1	41	-	Solar	55.6	2022	14
0.1	112.9	-	Solar	37.5	2022	15
0.1	287	-	Solar	26.2	2022	16
0.45	-	1.5	980 nm laser	5.0*	2022	17
0.3	330	-	Solar	27.5*	2023	18
0.1	130	-	Solar	24.5*	2023	19
0.1	273.9	-	Solar	60.4*	2023	20

0.1	149.5	-	Solar	20.0*	2023	[21]
0.002	-	1.15	1550 nm laser	3.1	2024	[22]
0.25	166.69	-	Solar	42.0	2024	[23]
6.37	-	13.53	1064 nm laser	196.4	2024	[24]
0.1	183	-	Solar	15.4	2024	[25]
0.1	14.5	-	950 nm laser	17.1	2024	[26]
**0.45**	**500.27**	-	**3000 K lamp**	**30.0**	**2025**	**This work**

*: Estimated ΔT from the data presented in the paper.

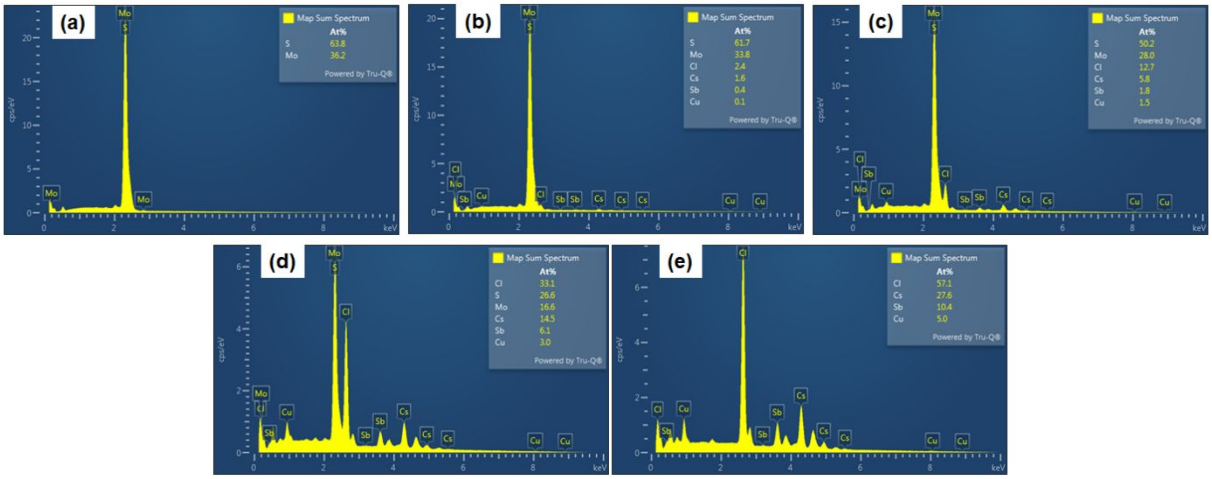

Figure S1. SEM-EDS of the respective samples of (a) pure MoS_2 films, and those with (b) 25, (c) 50, (d) 75 , and (e) 100 % CCSC film.

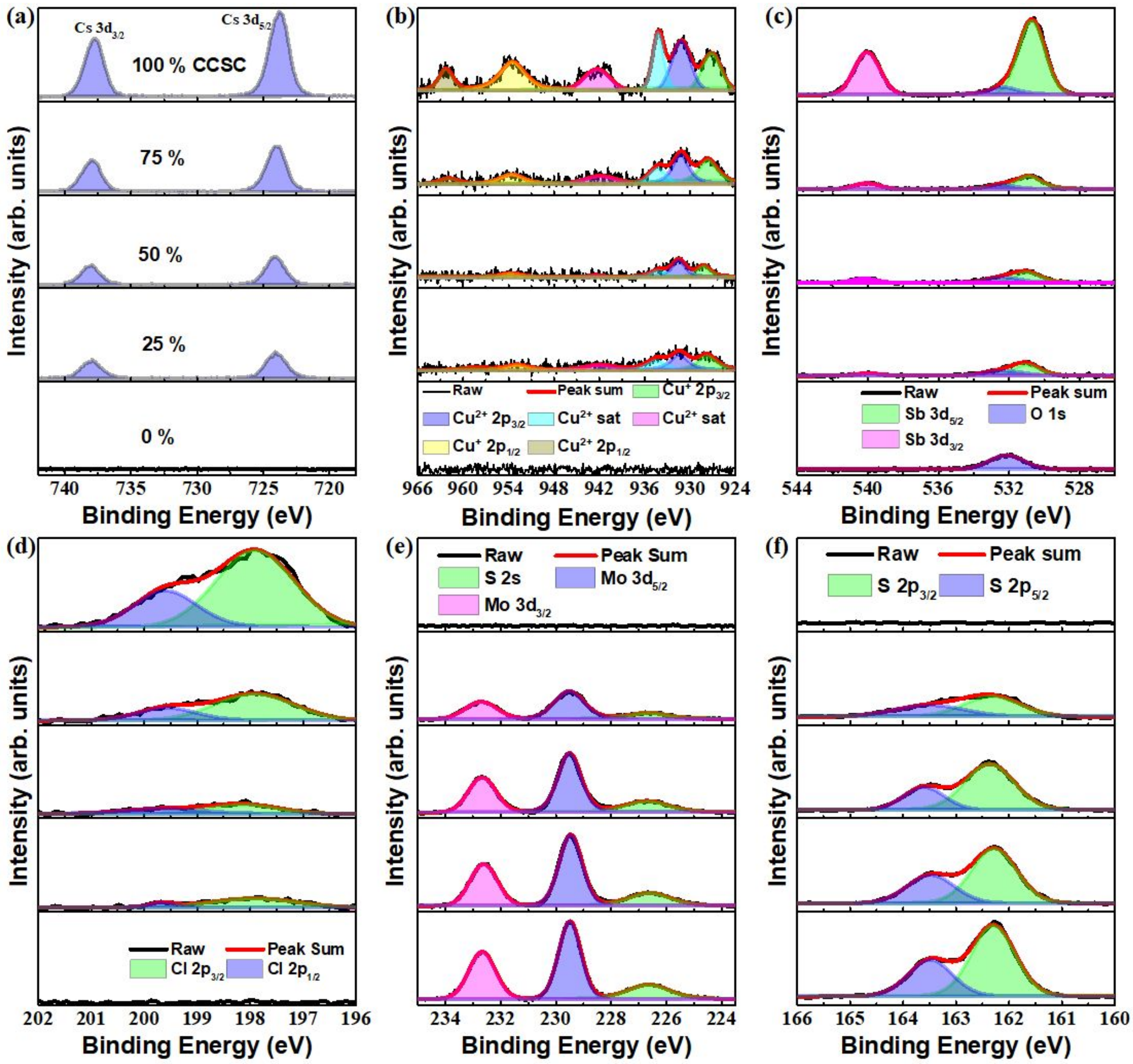

Figure S2. Chemical properties of CCSC/MoS_2 films. XPS scans for (a) Cs 3d, (b) Cu 2p, (c) Sb 3d, (d) Cl 2p, (e) Mo 3d, and (f) S 2p. The spectra were calibrated using C 1s peak, and each subfigure has the same scale for all the panels. The panels correspond to spectra obtained for the CCSC/MoS_2 samples with increasing CCSC concentration (from bottom to top) as represented in (a).

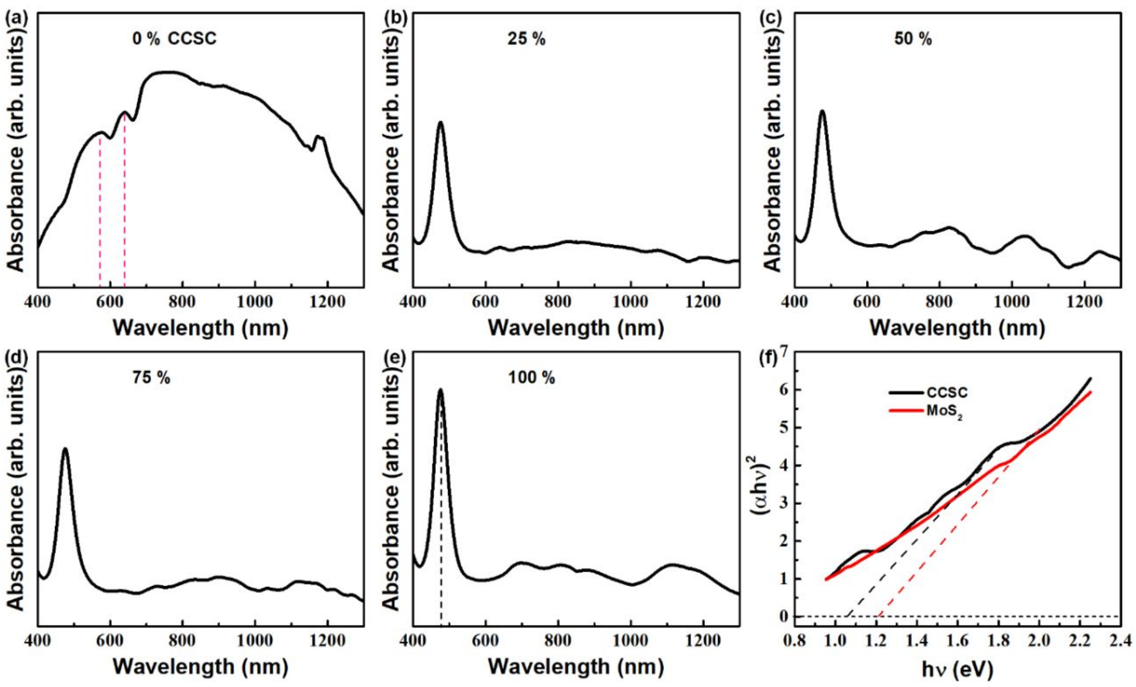

Figure S3. Optical absorption of the CCSC/MoS_2 composite. Absorption spectra of various composite films (on glass) with (a) 0, (b) 25, (c) 50, (d) 75), and (e) 100 % CCSC wt. %. (f) Tauc's plot of bulk MoS_2 and CCCS using data from (a) and (e) showing respective bandgaps for the pure films. The MoS_2 and CCSC absorption peaks in (a) and (e) are indicated using dashed lines.

The optical properties of the CCSC/MoS_2 composite films were characterized by UV-vis absorption spectroscopy (Figure S3a-e). The bulk MoS_2 sample showed standard peaks at 590 and 650 nm (Figure S3a).[27] CCSC showed a strong absorption peak at ~ 480 nm, which was reported previously (~2.55 eV) (Figure S3e).[28] The absorption in the composite films are dominated by the CCSC absorption near 480 nm (Figure S3b-d). The overall absorption intensity of the 0 % CCSC (bulk MoS_2) was found to be lower than the 100 % CCSC sample (Figure S3e). The 100 % CCSC sample had a higher absorption intensity in the NIR region compared to the pure MoS_2 (0 % CCSC) sample. The bandgap of the pure CCSC and bulk MoS_2 have been determined to be 1.07 and 1.22 eV, respectively, using Tauc's plot (Figure S3f), matching with previous reports.[28-30]

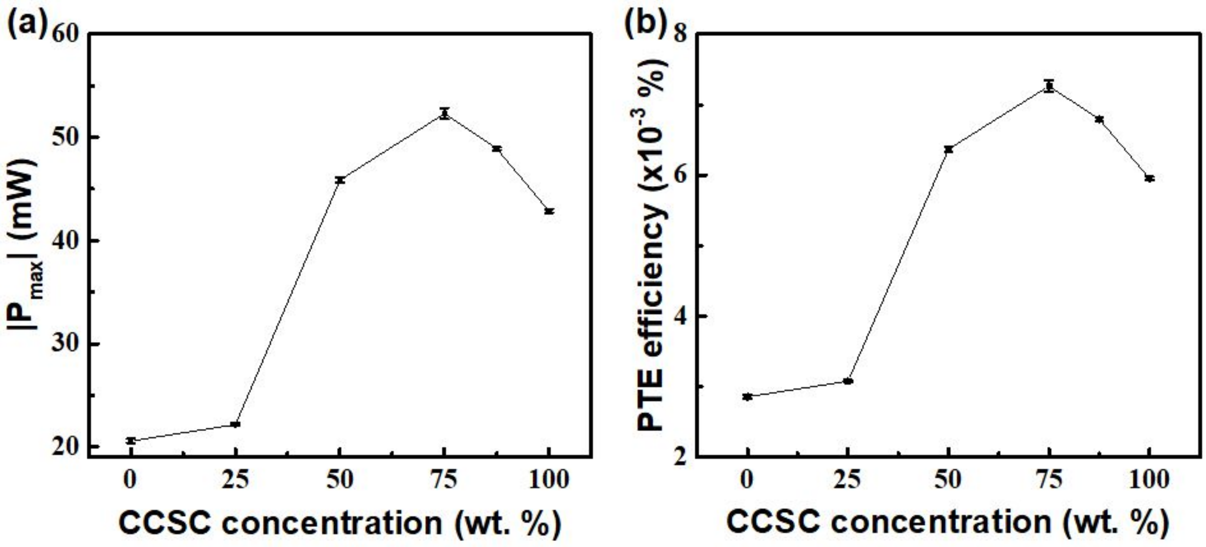

Figure S4. PTE **p**erformance parameters of the CCSC/MoS_2 composite coated films under 3000 K lamp. Estimated (a) PTE $|P_{max}|$, and (b) PTE efficiency of the various CCSC/MoS_2 composite coated PTE devices under 3000 K lamp (450 mW cm^{-2}). The error bars indicate the scatter of the data over 3 independent measurements. The lines joining the data points are guides to the eye only.

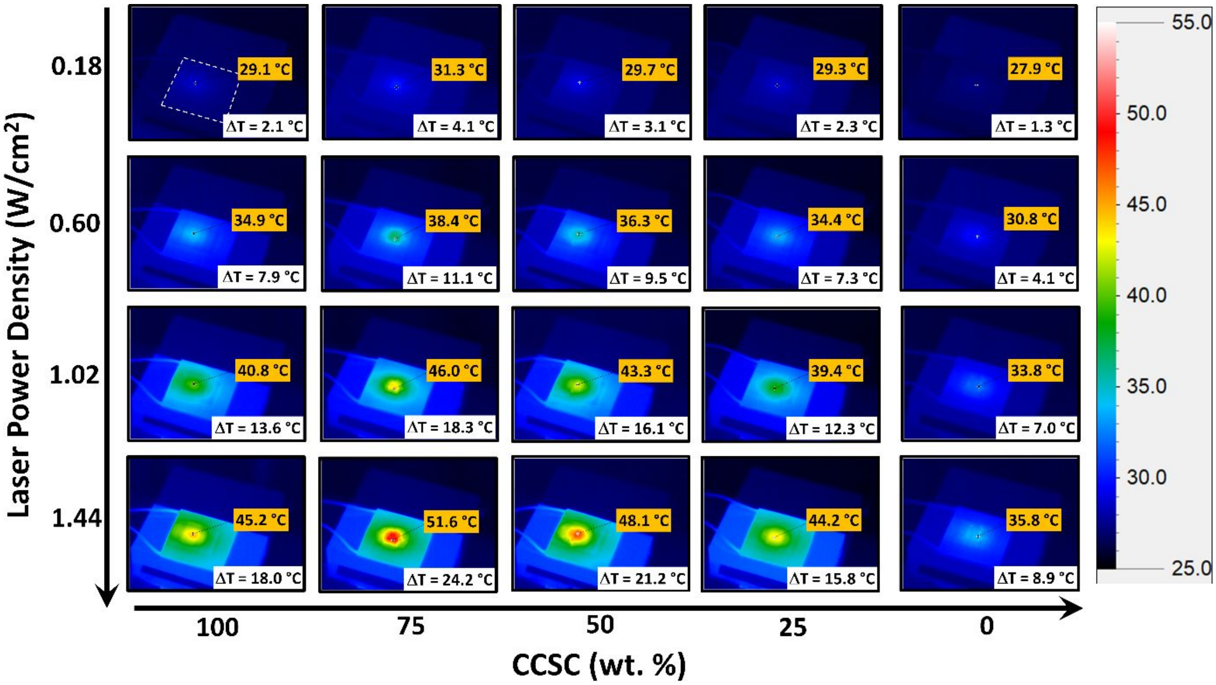

Figure S5. The power-dependent photothermal (PT) imaging of different CCSC/MoS_2 photo-thermo-electric (PTE) devices under 808 nm laser. The actual temperatures at incidence (arrowhead) are displayed in yellow boxes. The temperature difference (ΔT) from laser ON to OFF condition is indicated in white boxes. The temperatures are color-coded. The device area is outlined.

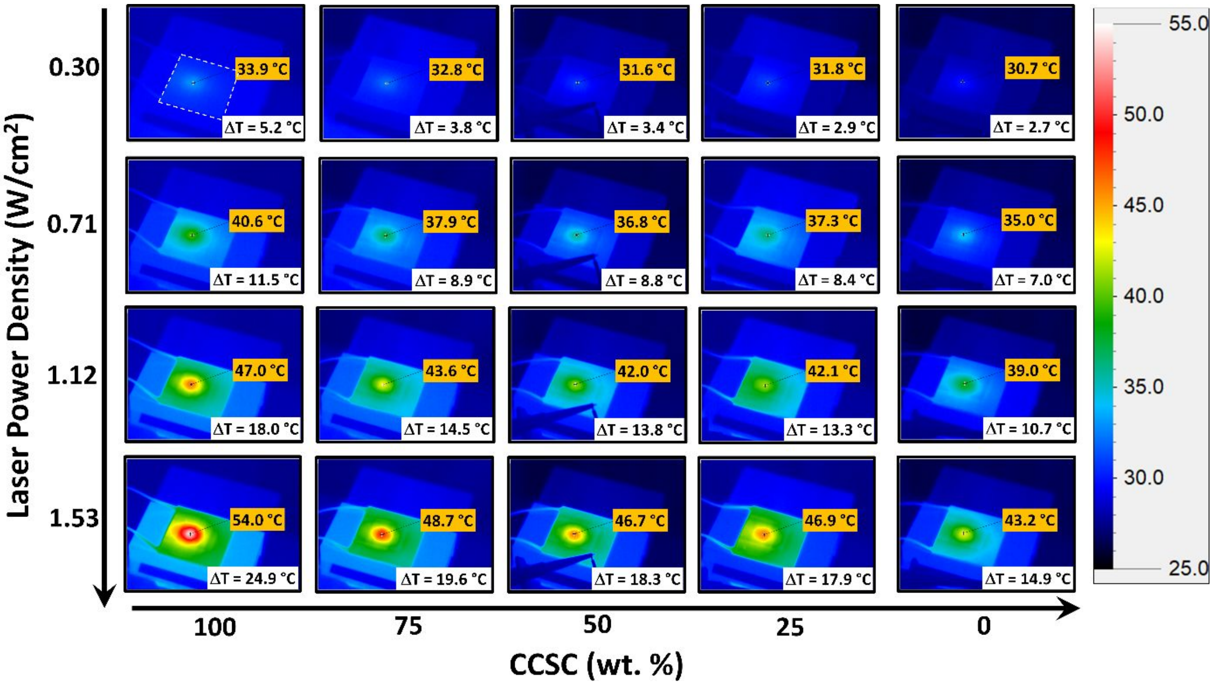

Figure S6. The power-dependent photothermal (PT) imaging of different CCSC/MoS_2 photo-thermo-electric (PTE) devices under 980 nm laser. The actual temperatures at incidence (arrowhead) are displayed in yellow boxes. The temperature difference (ΔT) from laser ON to OFF condition is indicated in white boxes. The temperatures are color-coded. The device area is outlined.

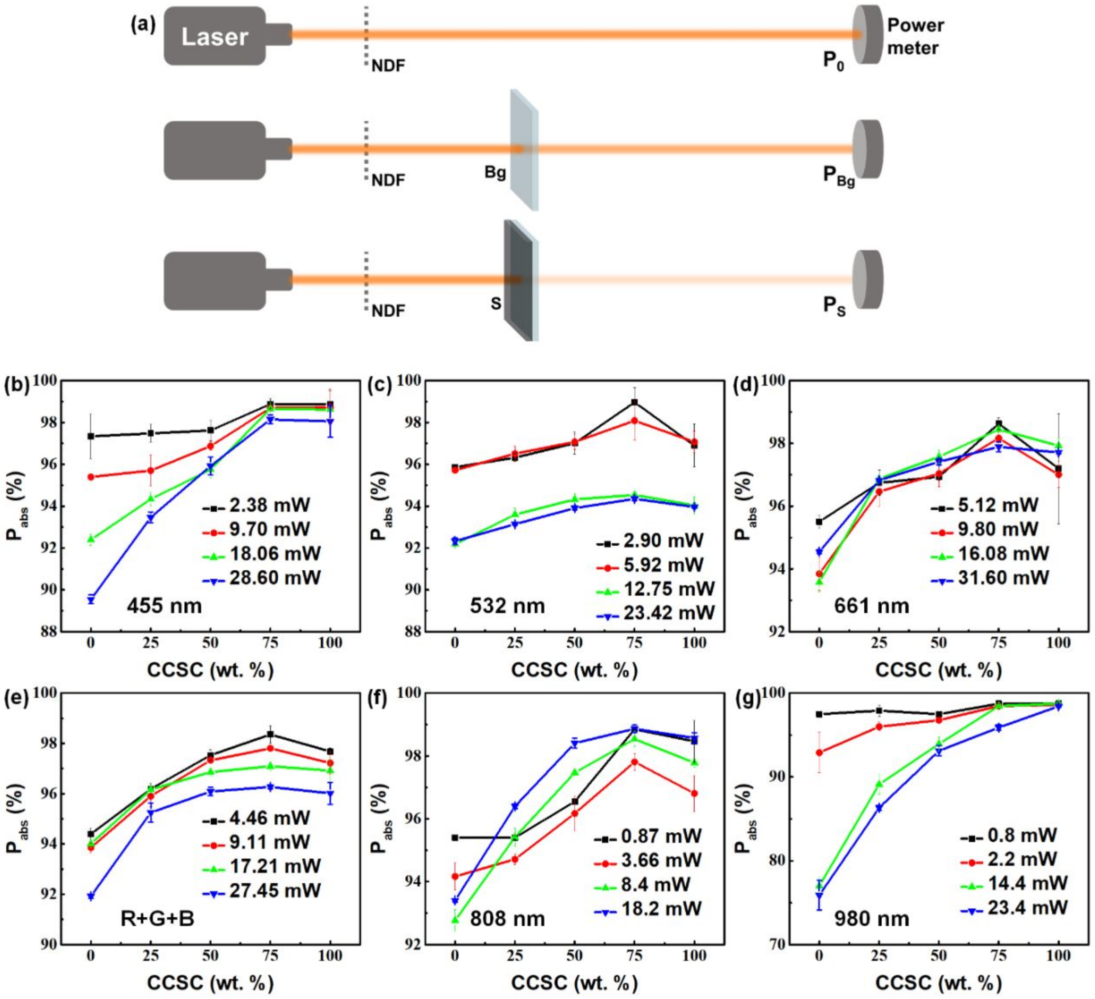

Figure S7. Optical power absorption of the CCSC/MoS_2 composite coated on glass under various laser illuminations. (a) Schematic of the transmitted power measurement setup using different lasers, and ND-filter (NDF), with glass as the background (Bg), and the sample (S) coated on glass. The incident power of the laser (P_0), that through the glass (P_{Bg}) and sample (P_S) are measured using a detector. Power-dependent absorption (P_{abs}) of the films with various CCSC wt. % under (a) 455 (B), (b) 532 (G), (c) 661 (R), (d) White (R+G+B), (e) 808, and (f) 980 nm laser illumination. The P_{abs} values were obtained from the power transmission setup (a). The measured P_{Bg} values were used for background correction. The line joining the data points is a guide to the eye only. Error bars indicate scatter over three independent measurements.

Figure S7 (a) shows the schematic of the experimental setup for measurement of absolute absorbed powers using laser illumination only. The 808 and 980 nm lasers were collimated over a circular beam of 1 cm diameter. The incident (P_0), background (P_{Bg}) and sample (P_S) transmitted powers were measured directly using a power-meter. The transmitted power % is calculated as follows

$$T\% = \frac{P_S - P_{Bg}}{P_0} \quad \text{S(1)}$$

From the above T %, the P_{abs} is calculated as

$$P_{abs}(\%) = 1 - T\% \quad \text{S(2)}$$

P_{abs} is then plotted for the films with varying CCSC wt. % under different illumination under different power densities (Figure S7b-g). here, a combination of R+B+G is used to simulate white light (Figure S7e). All the variations (Figure S7 b-f) show a clear P_{abs} maxima for the 75% CCSC film, which supports the highest PTE output for the 75 wt. % composite film. Only for the 980 nm illumination, a concomitant increase in P_{abs} was observed upto the 100 wt. % film as does the PTE outputs.

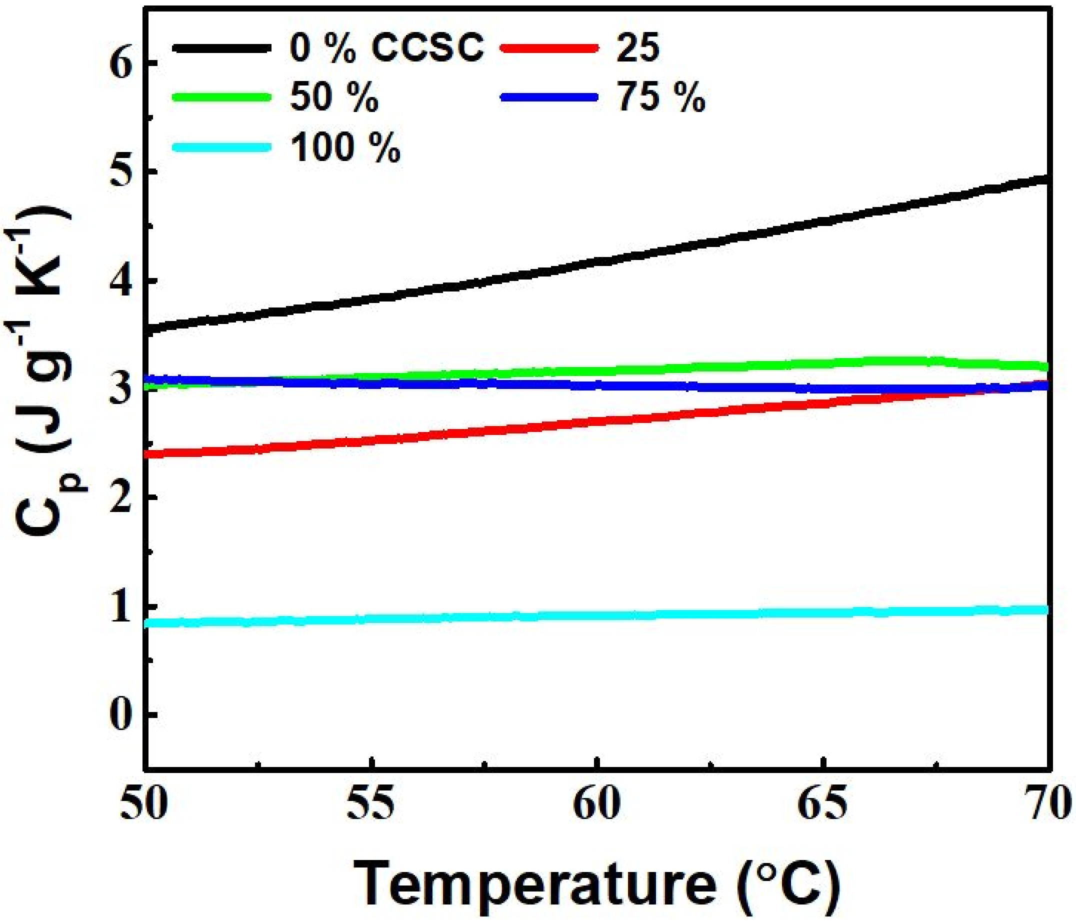

Figure S8. Thermal studies of MoS_2/CCSC films. Specific heat capacity (C_p) of the various MoS_2/CCSC films calculated from the differential scanning calorimetric (DSC) measurements.

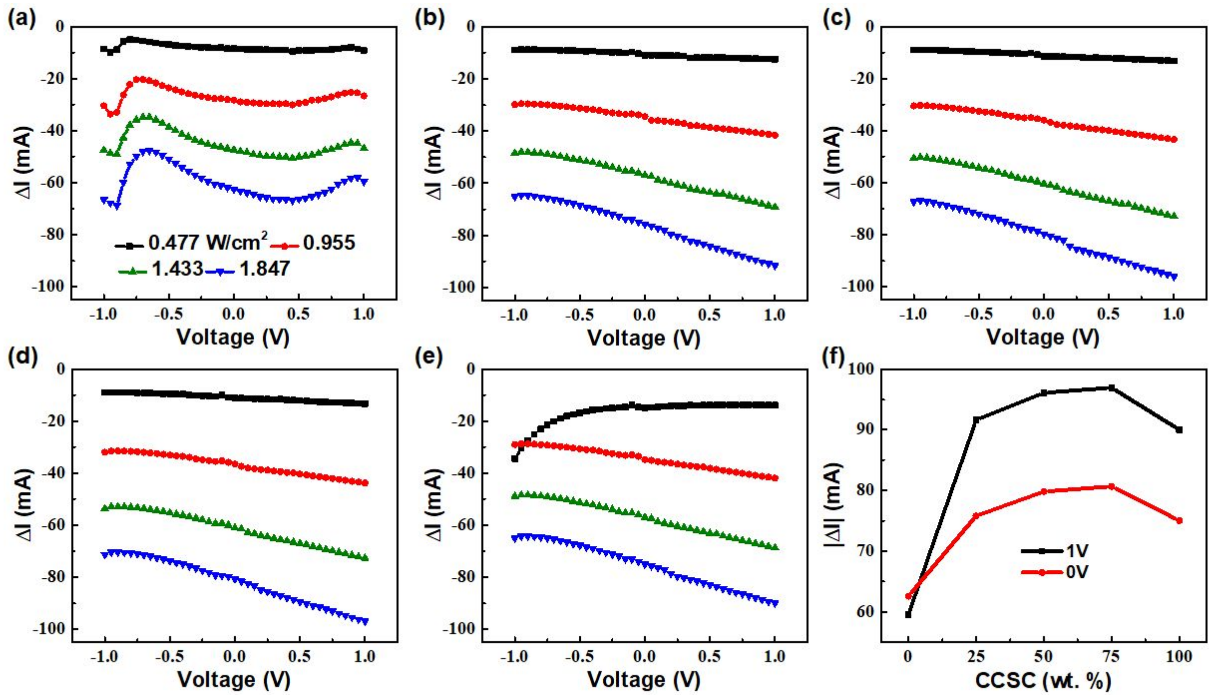

Figure S9. ΔI-V studies of PTE devices under 808 nm laser. ΔI-V of (a) 0 (pure MoS_2), (b) 25, (c) 50, (d) 75, and (e) 100 % (pure CCSC) CCSC/MoS_2 composite coated PTE devices under power-controlled (colour coded) 808 nm laser. (f) The comparison of ΔI with various CCSC/MoS_2 composite ratios at 0, and 1 V, respectively, under 1.44 W cm^{-2} of the 808 nm laser.

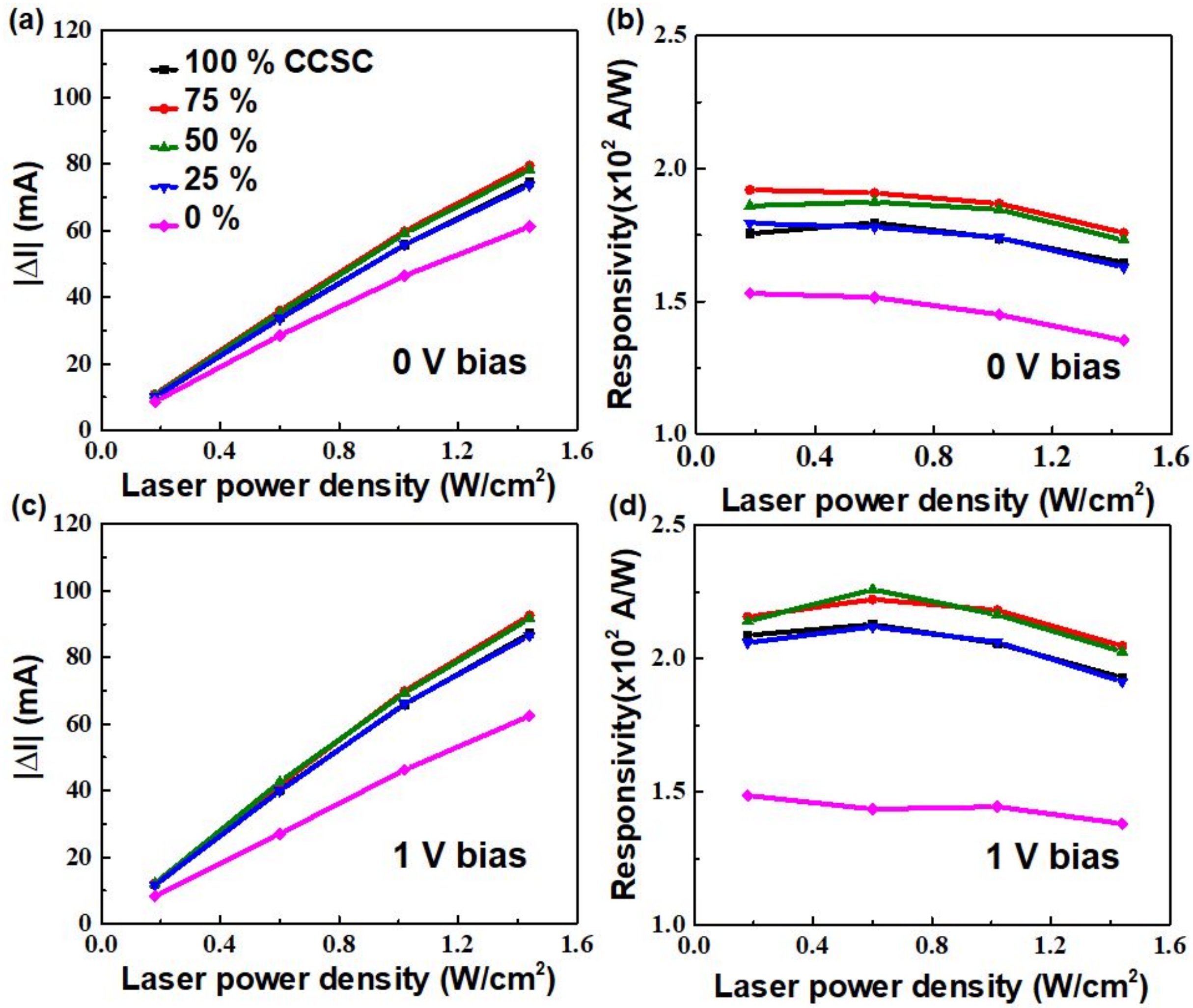

Figure S10. PTE performance parameters under 808 nm laser. PTE studies under 808 nm laser with an external bias of (a-b) 0, and (c-d) 1 V. Power-dependent photocurrent with a bias of (a) 0, (c) 1 V, and responsivity with a bias of (b) 0, (d) 1 V for various CCSC/MoS_2 composite (colour coded) coated PTE devices, respectively.

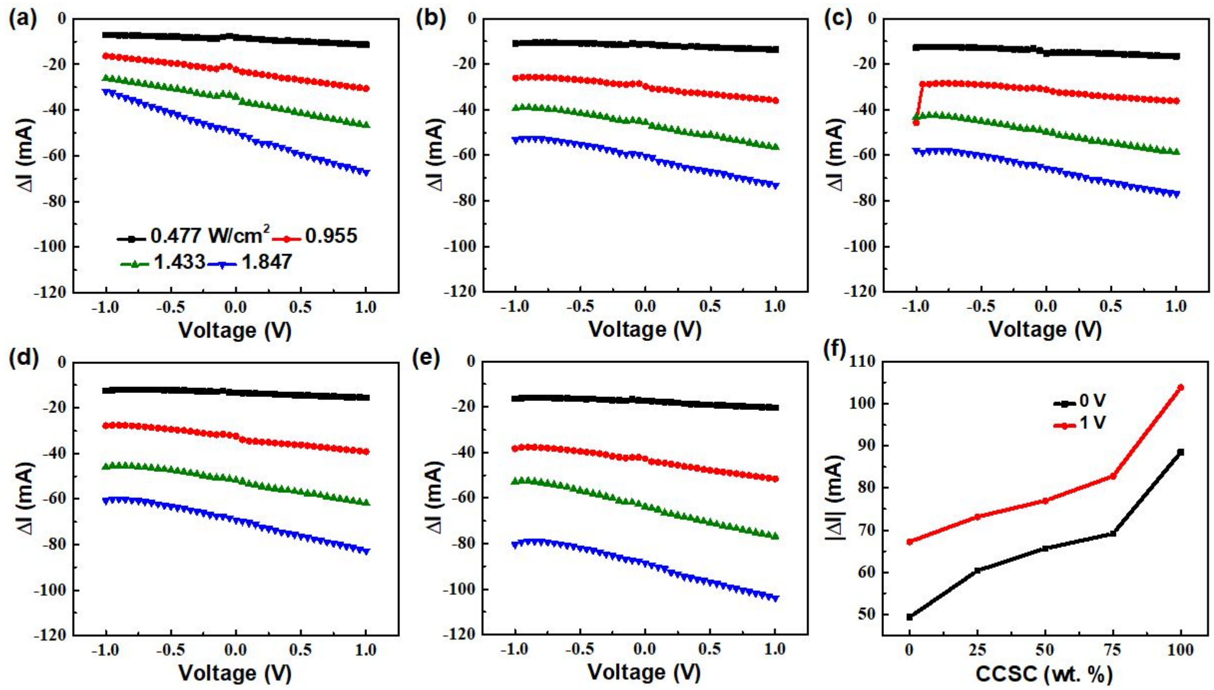

Figure S11. ΔI-V studies of PTE devices under 980 nm laser. ΔI-V of (a) 0 (pure MoS_2), (b) 25, (c) 50, (d) 75, and (e) 100 % (pure CCSC) CCSC/MoS_2 composite coated PTE devices under power-controlled (colour coded) 980 nm laser. (f) The comparison of ΔI with various CCSC/MoS_2 composite ratios at 0, and 1 V, respectively, under 1.53 W cm^{-2} of the 980 nm laser.

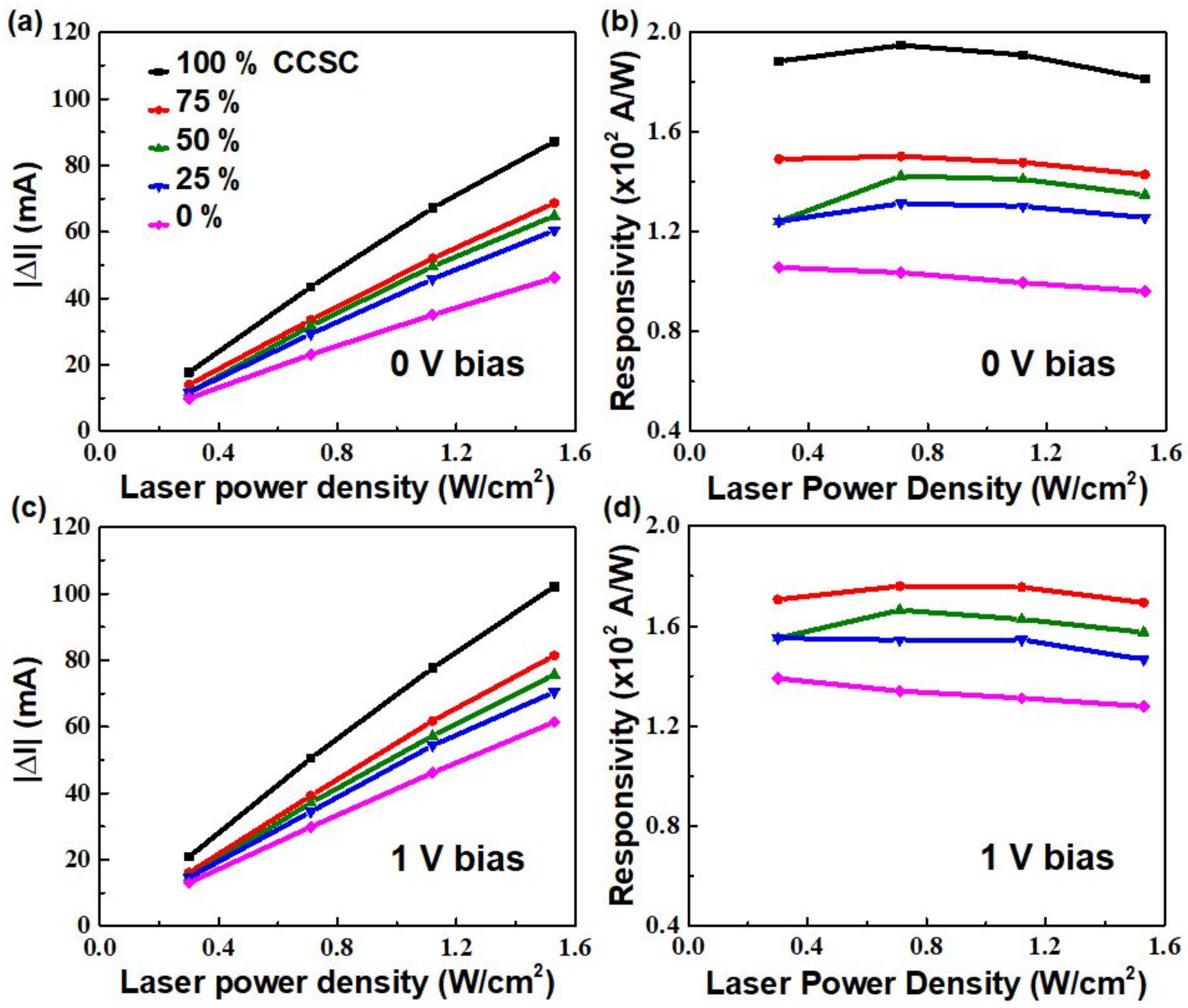

Figure S12. PTE performance parameters under 980 nm laser. PTE studies under 980 nm laser with an external bias of (a-b) 0, and (c-d) 1 V. Power-dependent photocurrent with a bias of (a) 0, (c) 1 V, and responsivity with a bias of (b) 0, (d) 1 V for various CCSC/MoS_2 composite (colour coded) coated PTE devices, respectively.

References (for ESI only)

1. Lee, D. H.; Pyun, S. B.; Bae, Y.; Kang, D. P.; Park, J.-W.; Cho, E. C., Solution-processed plasmonic–dielectric sunlight-collecting nanofilms for solar thermoelectric application. *ACS applied materials & interfaces* **2017,** *9* (50), 43583-43595.
2. Jung, Y. S.; Jeong, D. H.; Kang, S. B.; Kim, F.; Jeong, M. H.; Lee, K.-S.; Son, J. S.; Baik, J. M.; Kim, J.-S.; Choi, K. J., Wearable solar thermoelectric generator driven by unprecedentedly high temperature difference. *Nano energy* **2017,** *40*, 663-672.
3. He, M.; Lin, Y.-J.; Chiu, C.-M.; Yang, W.; Zhang, B.; Yun, D.; Xie, Y.; Lin, Z.-H., A flexible photo-thermoelectric nanogenerator based on MoS_2/PU photothermal layer for infrared light harvesting. *Nano Energy* **2018,** *49*, 588-595.
4. Hazama, H.; Masuoka, Y.; Suzumura, A.; Matsubara, M.; Tajima, S.; Asahi, R., Cylindrical thermoelectric generator with water heating system for high solar energy conversion efficiency. *Applied energy* **2018,** *226*, 381-388.
5. Zhang, X.; Gao, W.; Su, X.; Wang, F.; Liu, B.; Wang, J.-J.; Liu, H.; Sang, Y., Conversion of solar power to chemical energy based on carbon nanoparticle modified photo-thermoelectric generator and electrochemical water splitting system. *Nano Energy* **2018,** *48*, 481-488.
6. Wang, K.; Hou, Y.; Poudel, B.; Yang, D.; Jiang, Y.; Kang, M. G.; Wang, K.; Wu, C.; Priya, S., Melanin–perovskite composites for photothermal conversion. *Advanced Energy Materials* **2019,** *9* (37), 1901753.
7. Zhang, Y.; Umair, M. M.; Jin, X.; Lu, R.; Zhang, S.; Tang, B., An intelligent light-driven thermoelectric conversion system through the thermosensitive phase transition of vanadium dioxide. *Journal of Materials Chemistry A* **2019,** *7* (14), 8521-8526.
8. Xia, Z.; Zhang, Z.; Meng, Z.; Yu, Z., A 24-hour thermoelectric generator simultaneous using solar heat energy and space cold energy. *Journal of Quantitative Spectroscopy and Radiative Transfer* **2020,** *251*, 107038.
9. Shan, X.; Zhao, A.; Lin, Y.; Hu, Y.; Di, Y.; Liu, C.; Gan, Z., Low-cost, scalable, and reusable photothermal layers for highly efficient solar steam generation and versatile energy conversion. *Advanced Sustainable Systems* **2020,** *4* (5), 1900153.
10. Li, M.; Chen, J.; Luo, M.; Zhong, W.; Wang, W.; Qing, X.; Lu, Y.; Yang, L.; Liu, Q.; Wang, Y., Wearable thermoelectric 3D spacer fabric containing a photothermal ZrC layer with improved power generation efficiency. *Energy Conversion and Management* **2021,** *243*, 114432.
11. Xin, C.; Hu, Z.; Fang, Z.; Chaudhary, M.; Xiang, H.; Xu, X.; Aigouy, L.; Chen, Z., Flexible and wearable plasmonic-enabled organic/inorganic hybrid photothermoelectric generators. *Materials today energy* **2021,** *22*, 100859.
12. Cheng, P.; Wang, H.; Müller, B. r.; Müller, J.; Wang, D.; Schaaf, P., Photo-thermoelectric conversion using black silicon with enhanced light trapping performance far beyond the band edge absorption. *ACS Applied Materials & Interfaces* **2021,** *13* (1), 1818-1826.
13. Yang, Z.-y.; Jin, X.-z.; Wang, W.-y.; Huang, C.-h.; Lei, Y.-z.; Wang, Y., Ag 2 Se/nylon self-supporting composite films for wearable photo-thermoelectric generators with high output characteristics. *Journal of Materials Chemistry A* **2022,** *10* (39), 21080-21092.
14. Wu, Y.; Li, Y.; Long, Y.; Xu, Y.; Yang, J.; Zhu, H.; Liu, T.; Shi, G., High-efficiency photo-thermo-electric system with waste heat utilization and energy storage. *ACS Applied Materials & Interfaces* **2022,** *14* (35), 40437-40446.

15. Zhao, X.; He, Z.; Ou, W.; Lin, P.; Chen, Y.; Chen, Y., Narrow-bandgap light-absorbing conjugated polybenzobisthiazole: Massive interfacial synthesis, robust solar-thermal evaporation and thermoelectric power generation. *Science China Materials* **2022,** *65* (9), 2491-2501.
16. Tang, L.-S.; Zhou, Y.-C.; Zhou, L.; Yang, J.; Bai, L.; Bao, R.-Y.; Liu, Z.-Y.; Yang, M.-B.; Yang, W., Double-layered and shape-stabilized phase change materials with enhanced thermal conduction and reversible thermochromism for solar thermoelectric power generation. *Chemical Engineering Journal* **2022,** *430*, 132773.
17. Gu, Y.; Yao, X.; Geng, H.; Long, M.; Guan, G.; Hu, M.; Han, M., Large-area, flexible, and dual-source co-evaporated $Cs_3Cu_2I_5$ nanolayer to construct ultra-broadband photothermoelectric detector from visible to terahertz. *ACS Applied Electronic Materials* **2022,** *4* (2), 663-671.
18. Wang, H.; Shi, Y.; Liu, T.; Zheng, X.; Gao, S.; Lu, J., "One stone two birds" or "you can't have your cake and eat it too"? Effects of device dimensions and position of the thermoelectric module on simultaneous solar-driven water evaporation and thermoelectric generation. *Journal of Materials Chemistry A* **2023,** *11* (1), 419-433.
19. Cheng, P.; Wang, D., Easily Repairable and High-Performance Carbon Nanostructure Absorber for Solar Photothermoelectric Conversion and Photothermal Water Evaporation. *ACS Applied Materials & Interfaces* **2023,** *15* (6), 8761-8769.
20. Devinder, S.; Vishwakarma, M. K.; Joseph, S.; Pandey, S.; Joseph, J., UV-LIG-based perfect broadband absorber for solar thermoelectric generation. *ACS Applied Energy Materials* **2023,** *6* (10), 5616-5627.
21. Li, L.; Li, M.; Qin, Y.; Chen, Y.; Dai, W.; Zhang, Z.; Kong, X.; Gong, P.; Wang, Y.; Yang, R., Eicosane-based thermo-conductive phase change composite for efficient capture solar energy and using in real-environment as power source. *Chemical Engineering Journal* **2023,** *462*, 142273.
22. Zhang, M.; Liu, Y.; Guo, F.; Zhang, B.; Hu, B.; Li, S.; Yu, W.; Hao, L., High-Performance Flexible Broadband Photothermoelectric Photodetectors Based on Tellurium Films. *ACS Applied Materials & Interfaces* **2024,** *16* (5), 6152-6161.
23. Long, Y.; Li, X.; Li, Y.; Wang, L.; Zhu, H.; Shi, G., Hygroscopic assisted solar photo-thermal-electric conversion system for all-day power generation and daytime water collection. *Chemical Engineering Journal* **2024**, 152615.
24. Zheng, Y.; Li, X.; Zhou, J.; Qin, Y.; Deng, Y.; Wang, Y., Boosted photothermoelectric effect in silver nanoparticles decorated carbon nanotube films for infrared detection and actuation. *Carbon* **2024,** *219*, 118810.
25. Ma, P.; Wang, Y.; Zhang, X.; Yang, B.; Lang, J.; Yang, J.; Wang, Z.; Abdukayumb, A.; Hu, G., Photothermal conversion-enhanced thermoelectric generators combined with supercapacitors: An efficacious approach to integrated power generation and storage. *Chemical Engineering Journal* **2024,** *492*, 152406.
26. Zhao, Z.; Zhang, Z.; Guo, R.; Shi, W.; Zhang, Q.; Guo, Y.; Wang, Y.; Liu, D.; Xue, C., Enhanced photo-thermoelectric detection in black silicon with chimney-like texture. *Materials Today Communications* **2024,** *40*, 109723.
27. Kabel, J.; Sharma, S.; Acharya, A.; Zhang, D.; Yap, Y. K., Molybdenum disulfide quantum dots: properties, synthesis, and applications. *C* **2021,** *7* (2), 45.

28. Singhal, N.; Chakraborty, R.; Ghosh, P.; Nag, A., Low-bandgap $Cs_4CuSb_2Cl_{12}$ layered double perovskite: Synthesis, reversible thermal changes, and magnetic interaction. *Chemistry–An Asian Journal* **2018,** *13* (16), 2085-2092.
29. Berg, L.; Czack, G.; Gras, D.; Koch-Bienemann, E., *Gmelin handbook of inorganic and organometallic chemistry*. Springer: Berlin, Germany: 1993.
30. Lu, J.; Chen, M.; Dong, L.; Cai, L.; Zhao, M.; Wang, Q.; Li, J., Molybdenum disulfide nanosheets: From exfoliation preparation to biosensing and cancer therapy applications. *Colloids and Surfaces B: Biointerfaces* **2020,** *194*, 111162.
